# Analysis of Drought-Induced Proteomic and Metabolomic Changes in Barley (*Hordeum vulgare* L.) Leaves and Roots Unravels Some Aspects of Biochemical Mechanisms Involved in Drought Tolerance

**DOI:** 10.3389/fpls.2016.01108

**Published:** 2016-07-26

**Authors:** Klaudia Chmielewska, Paweł Rodziewicz, Barbara Swarcewicz, Aneta Sawikowska, Paweł Krajewski, Łukasz Marczak, Danuta Ciesiołka, Anetta Kuczyńska, Krzysztof Mikołajczak, Piotr Ogrodowicz, Karolina Krystkowiak, Maria Surma, Tadeusz Adamski, Paweł Bednarek, Maciej Stobiecki

**Affiliations:** ^1^Institute of Bioorganic Chemistry – Polish Academy of Sciences, PoznańPoland; ^2^Institute of Plant Genetics – Polish Academy of Sciences, PoznańPoland

**Keywords:** abiotic stress, barley, drought stress, mass spectrometry, metabolome, proteome

## Abstract

In this study, proteomic and metabolomic changes in leaves and roots of two barley (*Hordeum vulgare* L.) genotypes, with contrasting drought tolerance, subjected to water deficit were investigated. Our two-dimensional electrophoresis (2D-PAGE) combined with matrix-assisted laser desorption time of flight mass spectrometry (MALDI-TOF and MALDI-TOF/TOF) analyses revealed 121 drought-responsive proteins in leaves and 182 in roots of both genotypes. Many of the identified drought-responsive proteins were associated with processes that are typically severely affected during water deficit, including photosynthesis and carbon metabolism. However, the highest number of identified leaf and root proteins represented general defense mechanisms. In addition, changes in the accumulation of proteins that represent processes formerly unassociated with drought response, e.g., phenylpropanoid metabolism, were also identified. Our tandem gas chromatography – time of flight mass spectrometry (GC/MS TOF) analyses revealed approximately 100 drought-affected low molecular weight compounds representing various metabolite types with amino acids being the most affected metabolite class. We compared the results from proteomic and metabolomic analyses to search for existing relationship between these two levels of molecular organization. We also uncovered organ specificity of the observed changes and revealed differences in the response to water deficit of drought susceptible and tolerant barley lines. Particularly, our results indicated that several of identified proteins and metabolites whose accumulation levels were increased with drought in the analyzed susceptible barley variety revealed elevated constitutive accumulation levels in the drought-resistant line. This may suggest that constitutive biochemical predisposition represents a better drought tolerance mechanism than inducible responses.

## Introduction

Water scarcity (physical and economic) is a problem faced by modern agriculture and has the greatest impact on reduced field production. This issue will remain a serious threat to world food security unless means to circumvent the impacts of water deficit is developed. Plants, as sessile organisms, have evolved a variety of mechanisms to confront challenges from adverse abiotic and biotic environmental factors. For example, during drought stress, to limit water loss through transpiration plants close their stomata, but as a side effect it leads to diminished internal CO_2_ concentration which eventually results in lower photosynthetic rate (Cornic, 2000). The lower CO_2_ fixation depletes glycerate-3-phosphate, which decreases NADPH use in the Calvin cycle. The result is lower level of NADP^+^, which is the primary electron acceptor in photosystem I (PSI); O_2_ reduction is accelerated which leads to excessive generation of reactive oxygen species (ROS; Asada, 2006; Murata et al., 2007; Chen and Murata, 2008). ROS, unlike atmospheric oxygen, can oxidize various biomolecules and internal cell structures, thereby causing cellular damage (Dat et al., 2000). During drought, plants may increase activities of ROS-scavenging pathways, including water-water and ascorbate–glutathione cycles and enzymes like superoxide dismutase and catalase (Mittler, 2002; Asada, 2006). Drought may also trigger molecular chaperone accumulation (Wang et al., 2004) and the overproduction of compatible organic solutes (Serraj and Sinclair, 2002). Glycine betaine (GB) and proline are two main plant osmolytes that accumulate in response to various abiotic stresses (Ashraf and Foolad, 2007).

Proteomics and metabolomics became powerful tools for analyzing plant reactions to various environmental stimuli. Especially comparative studies of genetically diverse germplasms subjected to adverse conditions like drought provide valuable insights into plant responses to pre-determined stress and give information of the biochemical pathways that participate in acclimation to environmental constrains. Proper evaluation of proteomic and metabolomic data can contribute to a process of biomarker discovery. In the case the potential candidates are successfully correlated with corresponding quantitative trait loci (QTLs), they can be further integrated into marker-assisted breeding strategy to enhance selection of plants with desired traits (Tuberosa and Salvi, 2006). Many studies were performed to determine the effects of drought and other abiotic stresses on crop plants at the proteomic and metabolomic level (Kopka et al., 2004; Riccardi et al., 2004; Cramer et al., 2007; Zuther et al., 2007; Caruso et al., 2009; Skirycz et al., 2010; Ford et al., 2011; Bowne et al., 2012; Witt et al., 2012). In these studies abundantly identified proteins and metabolites were usually involved in defense mechanisms, including detoxification enzymes, redox status regulation, signaling pathways, protein folding and degradation, photosynthesis, and primary metabolism.

Barley is the fourth most important cereal in total worldwide production after wheat, maize, and rice^1^. It is used for animal feed, malt production, and human food. The species is a good experimental model for studying cereal plant biology, primarily due to its short life cycle (~90 days), autogamous nature and relatively small diploid genome (5.3 Gbp), particularly when compared to hexaploid wheat (18 Gbp). Recently, the physical, genetic, and functional sequence of the barley genome was assembled (The International Barley Genome and Sequencing Consortium, 2012). Drought or salt susceptible and tolerant barley varieties were also recently examined under abiotic stresses and subjected to proteomic or metabolomic analyses (Ashoub et al., 2013). However there is a lack of publications that cover simultaneously the area of proteomic and metabolomic research performed in one analysis.

The aim of the present study was to evaluate with mass spectrometry techniques proteome and metabolome changes in leaves and roots of two barley (*Hordeum vulgare* L.) genotypes of different origin subjected to drought. The phenotypic traits measured at the harvest point indicated significant differences in the tolerance to water deficit between those two lines. This distinctive effect of drought on growth of both barley varieties allowed us to correlate some of the differences observed in their proteomes and metabolomes with enhanced drought resistance.

## Materials and Methods

### Plant Growth and Stress Treatment

Barley (*Hordeum vulgare* L.) plants were cultivated under partially controlled greenhouse conditions. Two spring varieties — Maresi and Cam/B1//CI08887/CI05761 (referred further as Cam/B1/CI) — were used for the experiments. Maresi is a German semi-dwarf variety and Cam/B1/CI is a Syrian breeding line; the two varieties were chosen based on the results on their tolerance to reduced water supply reported (Górny, 2001). Seeds of the Syrian genotype were kindly supplied by the International Center for Agricultural Research in the Dry Areas (ICARDA; Syria), and seeds of Maresi originated from the Plant Genetic Resources (Czech Republic).

The plants were grown in pots containing 8 kg of soil (loamy sand mixed with sand at a weight ratio of 7:2). Water properties of soil were characterized by water retention curve. Soil moisture at 2.2 pF (15.8 kPa) for control and 3.2 pF (158.5 kPa) for drought was established. The amount of added fertilizer was established on the basis of soil-tests. Each variety was grown in both control and drought conditions (16 pots in total). In each pot 10 plants were cultivated and when harvested treated as one pooled biological repetition. Half of the plant material was used for metabolomic and proteomic studies and the other half for phenotypic traits analysis. This experiment was repeated twice.

The drought began at the three-leaf stage (phase 13 in the BBCH scale), which was achieved 16 days after sowing and lasted 10 days. The soil moisture was controlled daily using an FOM/mts TDR soil moisture meter according to the reflectometry method (EasyTest, Institute of Agrophysics PAS, Poland) and adjusted by adding the appropriate quantity of water.

The plant material (leaves and roots) for proteomic and metabolomic analysis was collected directly after the 10-days drought period. The harvested samples were immediately frozen in liquid nitrogen. The tissue samples were ground in liquid nitrogen in precooled adaptors for 45 s at 30 Hz frequency using a ball mill MM400 (Retsch, Germany). Pulverized tissue was stored at -80°C until further analyses.

Plants used for the phenotypic traits analysis were grown after the drought period maturity under control conditions. Traits related to plant architecture, growth and productivity were measured on the basis of four replicates.

### Protein Extraction

The total protein from leaves and roots was extracted using the procedure described by Hurkman and Tanaka (1986) with certain modifications. In brief, homogenized tissue was weighed (300 and 600 mg of leaf and root samples, respectively) and suspended in 750 μl of cold lysis buffer [500 mM Tris-HCl pH 8.0; 700 mM saccharose; 100 mM KCl; 50 mM EDTA; 2 mM phenylmethylsulfonyl fluoride; and 2% (v/v) β-mercaptoethanol] and incubated for 10 min on ice. Subsequently, an equal volume of water-saturated phenol (750 μl) was added to each sample. The solutions were incubated on a shaker for 10 min at room temperature. The aqueous and organic phases were separated by centrifugation for 10 min at 11, 000 *g* at 4°C. The phenolic phase was recovered and re-extracted with an equal volume of extraction buffer. 5 volume of 0.1 M ammonium acetate in methanol was added to phenolic phase and the proteins were precipitated overnight in -20°C. The precipitated proteins were collected by centrifugation for 5 min at 18, 000 *g* and 4°C and then washed three times with 0.1 M ammonium acetate in methanol. One percent (w/v) polyvinylpyrrolidone (PVP) was added to the first wash to facilitate phenolic contamination removal. Finally, the precipitated proteins were washed with 80% acetone. The samples were centrifuged for 2 min at 18, 000 *g* and 4°C after each wash step and air dried.

### Metabolite Extraction

50 mg of frozen barley samples (leaves or roots) was suspended in 1.4 ml of 80% methanol. The ribitol was used as an internal standard (25 and 10 μl of 1.0 mg/ml solution for leaves and roots, respectively). The mixtures were shaken vigorously for 10 min at room temperature in a thermomixer (TS-100, Biosan, Latvia) at 950 rpm. The suspensions were centrifuged at 11, 000 *g* in RT. For leaf and root samples respectively, 200 and 500 μl aliquots of the supernatant was evaporated in RT using vacuum concentrator (Eppendorf, Germany). The dried extract samples were redissolved in 50 μl of 20 mg/ml methoxyamine hydrochloride solution in pyridine, and the derivatization reaction was performed for 1.5 h followed by a 30 min reaction with 80 μl of *N*-methyl-*N*-(trimethylsilyl)trifluoroacetamide (MSTFA). Both reactions were performed at 37°C. A mixture of C10–C36 alkanes in hexane was used as the retention index marker.

### 2D Gel Electrophoresis of the Proteins

Isoelectric focusing (IEF, first dimension) was performed by dissolving the total protein extracts in the rehydration buffer [7 M urea, 2 M thiourea, and 2% (w/v) CHAPS]. 2D Quant Kit (GE Healthcare, USA) was used to quantify the protein concentration. 300 μg of the protein sample was loaded onto an 11 cm Immobiline DryStrip with a linear gradient of pH 4–7 (GE Healthcare, USA). Isoelectric focusing (IEF) was performed using an ETTAN IPGphor 3 System (GE Healthcare, USA) according to the manual. The IPG strips were equilibrated twice for 15 min in 7.5 ml of equilibration buffer. The first equilibration solution contained 1.5 M Tris-HCl, pH 8.8, 6 M urea, 30% (v/v) glycerol, 2% (w/v) SDS, 0.002% (w/v) bromophenol blue, and 1% (w/v) dithiothreitol. The second equilibration solution was modified by replacing the dithiothreitol with 2.5% (w/v) iodoacetamide. In the second dimension proteins were separated in 12% SDS-polyacrylamide gels on Ettan DALTtwelve System (GE Healthcare, USA). SDS-PAGE was performed using the following conditions: the temperature was maintained at 19°C, and the wattage was 1.25 W per gel for 30 min and 7.5 W per gel for 2 h. Two technical replicates were conducted for each biological replicate; therefore, eight gels for the leaf samples and eight gels for the root samples were analyzed for each cultivar, giving in total 64 gels.

### Gel Image and Statistical Analysis

The gels were stained with Colloidal Coomassie Brilliant Blue (Neuhoff et al., 1985). ImageScanner III (GE Healthcare, USA) was used to scan the gels. Image Master 2D Platinum 7.0 software (GE Healthcare, USA) was used to quantitatively analyze the spots. The protein spots across the gels were matched automatically, however, manual edition was necessary to improve the analysis. Protein spots relative volume (% vol) was quantified. This parameter is relatively independent of variation due to protein loading and staining. Statistical significance of the relative change of accumulation of protein spots was determined using Student’s *t*- test.

### In-Gel Digestion and Protein Identification by Mass Spectrometry

Protein spots were excised from the gels and digested with trypsin following the protocol described by Shevchenko et al. (1996) with certain modifications. Each excised gel piece was rinsed for 15 min with 100 μl 50 mM ammonium bicarbonate/acetonitrile and for 15 min with 100 μl 50 mM ammonium bicarbonate. Subsequently, the gel pieces were dehydrated with 100 μl acetonitrile and dried under vacuum centrifugation. A trypsin solution (Promega, USA) was added to the dry gel pieces, and the samples were incubated overnight at 37°C. Next, 1 μl of acetonitrile was added to the gel pieces, and the samples were subjected to sonication for 5 min and centrifugation (10, 000 *g*, 1 min). Subsequently, the isolated peptides were analyzed using MALDI-TOF or MALDI-TOF/TOF mass spectrometers model Autoflex and UltrafleXtreme (Bruker Daltonics, Germany) respectively. The matrix used was α-cyano-4-hydroxycinnamic acid dissolved in 50% acetonitrile with addition of 0.1% of trifluoroacetic acid. Mass spectra were registered in reflectron positive ion mode. The peptide mass fingerprinting (PMF) data were submitted to databases using the MASCOT in-house server. For cases in which the PMF analysis did not yield suitable results, an MS/MS analysis was performed on five peaks selected from the PMF spectrum. Database queries were restricted to Green Plants (*Viridiplantae*) and monoisotopic peptide masses were search. The criteria applied to accept the results were based on the molecular weight search score (MOWSE), percent sequence coverage, and matched peptide numbers.

### Metabolite Profiling Using Gas Chromatography/Mass Spectrometry

Qualitative and quantitative analyses were performed using a 6890 N gas chromatograph (Agilent, USA) and a GCT Premier mass spectrometer (Waters, USA) with Waters MassLynx software version 4.1. Gas chromatography was performed using a DB-5MS capillary column (30 m × 0.25 mm with a 0.25 μm film thickness; J&W Scientific, USA). The injection temperature was set to 230°C, the MS transfer line was at 230°C, and the ion source was adjusted to 250°C. Pure helium was used as the carrier gas at a constant flow of 1 ml/min. The oven temperature was maintained at 70°C for 2 min, then ramped at 10°C/min to 300°C, and finally maintained at 300°C for 10 min. Mass spectra were recorded in the *m/z* range 50–650 with electron ionization (70 eV) in the positive ion mode. Each biological replicate was analyzed using four technical repetitions. The obtained mass spectra were analyzed by the TargetSearch software package (Cuadros-Inostroza et al., 2009), using the Golm Metabolome Database (GMD) as the reference library (Kopka et al., 2005).

Two peaks that originated from the oligosaccharides raffinose and 1-kestose were not fully resolved on the TIC due to the co-elution on the GC column and high similarity of mass spectra, which was also found during previous investigations (Bowne et al., 2012). This lack of resolution disabled automated assignment and alignment with TargetSearch. For this reason, manual integration of raffinose and 1-kestose peaks was performed.

### Statistical Data Analysis

The metabolomic and phenotypic data were submitted to an analysis of variance in Genstat 16 (VSN International, Ltd^2^) to identify significant mean differences among the varieties, between control and drought conditions, and to identify significant interaction of varieties with different conditions.

## Results

### Drought Influence on the Phenotypic Traits of Analyzed Barley Genotypes

**FIGURE 1:**
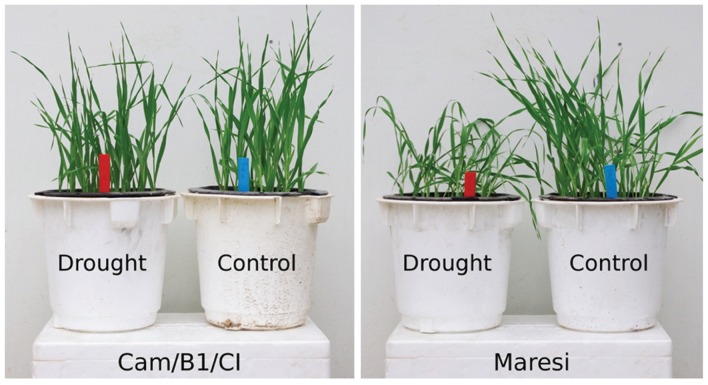
**Growth phenotypes of Maresi and Cam/B1/CI plants exposed to the 10-days long drought conditions**.

Studied genotypes clearly differed in response to the applied 10-days drought period in terms of maintaining turgor pressure (**Figure 1**). At this time point relative water content (RWC) was comparable in control plants of both lines tested barley lines (Maresi: 97 ± 2%; Cam/B1/CI: 94 ± 1%). However, the RWC measured in plants exposed to drought conditions indicated that Cam/B1/CI is less prone to water loss (Maresi: 75 ± 2%; Cam/B1/CI: 87 ± 3%). The results of phenotypic measurements performed for mature plants revealed several differences in drought impact on the performance of both tested barley lines. Significant effects (*P* < 0.05) for the drought effect were observed for eight traits in Maresi and for four traits in Cam/B1/CI, with one trait in common (**Table 1**). The relative reduction of yield in drought was similar for both varieties (about 30%), but the absolute loss for Maresi (*P* < 0.05) was about two times bigger than the loss for Cam/B1/CI. The two varieties attained the yield reduction in a different way. For Maresi, the lower yielding was due to a lower grain weight and number of grains on main spike, length of spikes and number of productive tillers. Cam/B1/CI reacted to drought by significantly reducing its 1000-grain weight and number of spikelets in lateral spikes. The protein content in seeds was significantly increased in drought only in Maresi. Overall, our results suggest that Cam/B1/CI is better adapted to drought conditions than Maresi.

**Table 1:** Drought induced changes in phenotypic traits observed in mature barley plants.

Group of traits	Trait	Control		Drought		Standard error	Drought impact	
		Maresi	Cam	Maresi	Cam		Maresi	Cam
Yield	1000-grain weight [g]	41.7	42.6	39.7	31.3*	1.7		↓
	Grain weight per plant [g]	2.7	1.7*	1.6	1.2	0.3	↓	
	Grain weight per main spike [g]	0.9	0.6*	0.7	0.4*	0.1	↓	
	Grain weight per lateral spike [g]	0.6	0.4*	0.5	0.3*	0.1		
Height, biomass	Length of main spike [cm]	7.4	6	5.9	4.8	0.4	↓	
	Length of lateral spike [cm]	6.0	4.6*	4.9	4.1	0.3	↓	
	Length of main stem [cm]	59.8	68.1	55	54.7	2.7		↓
	Straw weight per plant [g]	2.9	1.8	2.3	1.7	0.3		
Grain	Number of grains per main spike	18.8	12.6*	15.5	10.9*	0.7	↓	
	Number of grains per lateral spike	14.5	10.9*	13.2	8.7*	0.9		
Tillers	Number of productive tillers per plant	4.1	3.9	2.9	3.4	0.3	↓	
Protein	Protein content in grain [%]	13.6	16.3*	14.6	17.2*	0.2	↑	
Spikelets	Number of spikelets per main spike	19.2	14.4*	16.1	11.0*	0.6	↓	↓
	Number of spikelets per lateral spike	15.0	11.6*	14.6	9.9	0.4		↓

*Asterisks indicate a statistically significant difference between varieties (*P* < 0.05). Arrows indicate the direction of statistically significant drought-induced changes of the trait (*P* < 0.05, *n* = 4).*

### Drought-Induced Changes in the Barley Leaf and Root Proteomes and Metabolomes

**FIGURE 2:**
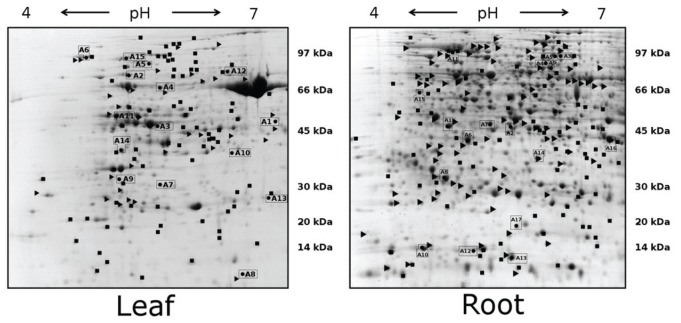
**Two-dimensional electrophoretic gels obtained during analysis of protein extracts from barley leaves and roots.** Protein samples were separated on an immobilized pH gradient strip, followed by SDS-PAGE. Labels indicate drought responsive proteins: (■) – Maresi; (►) – Cam/B1/CI. Spots enclosed in rectangles represent proteins whose accumulation levels changed following drought treatment in both tested barley genotypes.

**FIGURE 3:**
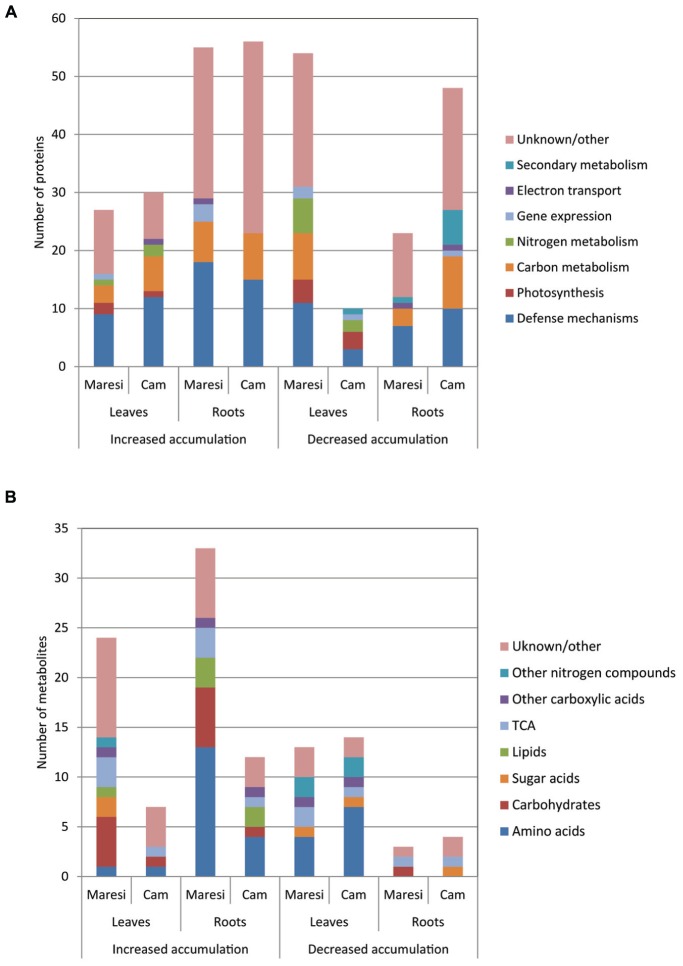
**Functional classification of drought-responsive proteins **(A)** and metabolites **(B)** identified in leaves and roots of Maresi and Cam/B1/CI plants**.

2D gel electrophoretic separation enabled reproducible detection of over 1000 protein spots (**Figure 2**; Supplementary Images S1–S4). The leaf proteome reaction to drought clearly differentiated tested genotypes. Prevalent number of all 81 drought-affected Maresi leaf proteins decreased their accumulation when compared to control (**Figure 3A**). In Cam/B1/CI leaves, only 40 proteins were influenced by drought conditions, but opposite to Maresi bigger fraction of them enhanced their accumulation (**Figure 3A**). In contrast to the leaf tissue, greater number (104) of root drought-responsive proteins was observed in Cam/B1/CI compared to 78 identified in Maresi. Also, a clearly higher number of proteins responded to stress by reducing their accumulation in Cam/B1/CI roots as compared to Maresi. We were able to identify ~70% of leaf and ~60% of root drought-responsive proteins by mass spectrometry and grouped them based on their function in biological processes (**Figure 3A**). The highest number of identified leaf and root proteins represented defense mechanisms and carbon metabolism respectively. In Cam/B1/CI leaves, opposite to Maresi, we did not find any proteins belonging to carbon metabolism category with reduced concentrations (**Figure 3A**). Accumulation levels of all proteins linked with gene expression identified in Cam/B1/CI were reduced in drought conditions, while in Maresi some representatives of this functional category increased their amounts in leaves and roots. Drought affected nitrogen metabolism in leaves, but not in roots of both tested barley lines. Finally, all identified enzymes linked with secondary metabolites reduced their accumulation in plants exposed to drought.

GC/MS analyses of leaf and root extracts enabled detection of 86 and 85 drought-affected metabolites respectively (**Figure 3B**, Supplementary Table S1). Primary metabolites constituted the main group among these compounds. Due to drought treatment significant (*P* < 0.01) changes in accumulation were observed for 23 leaf metabolites and 13 identified in roots in at least one variety. Mean drought effects were more frequent than interaction effects both in leaves (23/8) and roots (13/2). The metabolomic reaction to drought varied between the two studied genotypes with higher number of changes in metabolite accumulation observed in Maresi. There was also a clear difference between the total numbers of up-regulated, but not down-regulated, metabolites between tested genotypes (**Figure 3B**). 18 leaf and 21 root metabolites that were found to be up-regulated in Maresi did not change their accumulation levels in the same organs of Cam/B1/CI. Identified metabolites were grouped into respective metabolite classes. Among those the highest number of drought-responsive compounds represented amino acids (**Figure 3B**). Particularly response of Maresi roots to drought was linked with increased accumulation of 13 compounds representing this group. Interestingly, we did not find any amino acids whose accumulation was significantly reduced in roots of both tested barley lines.

### Defense Related Proteins

**FIGURE 4:**
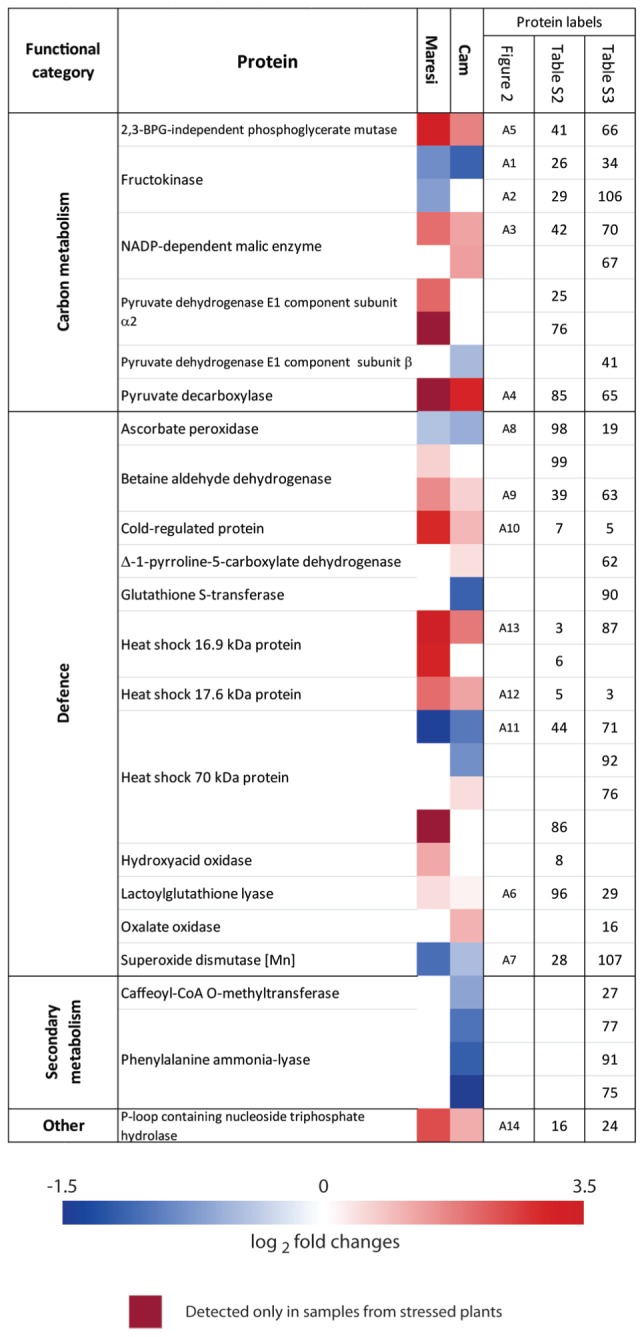
**Drought-induced changes in the accumulation levels of selected proteins identified in roots of both tested barley lines.** Additional data are shown in Supplementary Tables S2 and S3.

**FIGURE 5:**
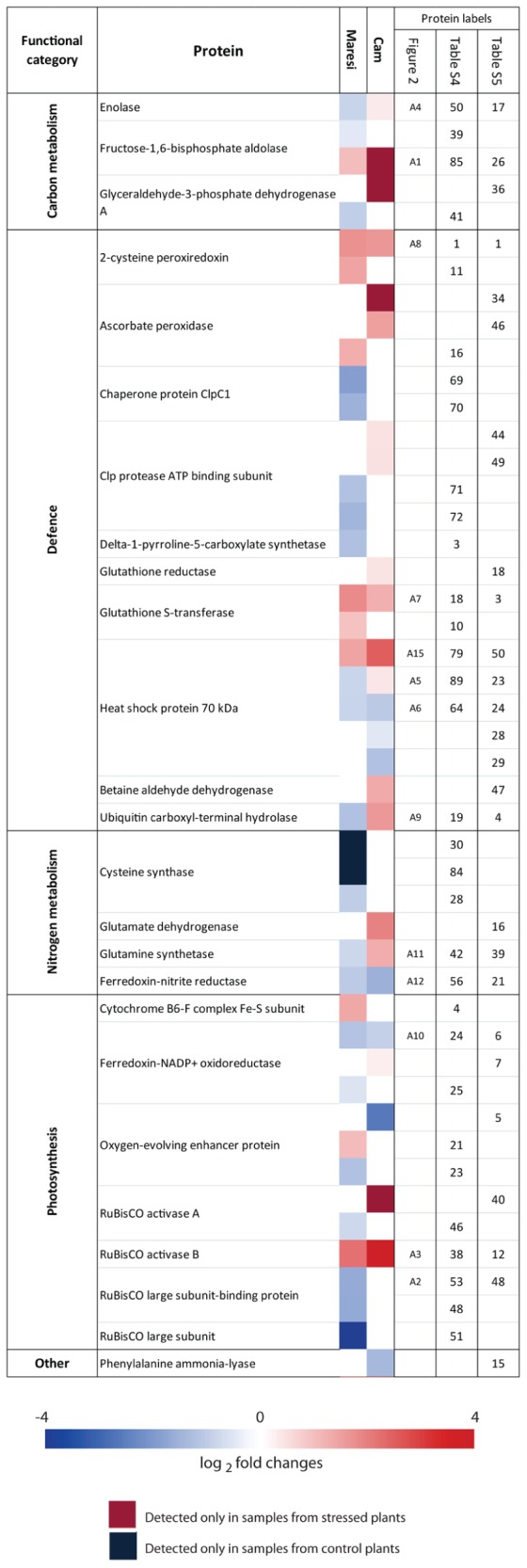
**Drought-induced changes in the accumulation levels of selected proteins identified in leaves of both tested barley lines.** Additional data are shown in Supplementary Tables S4 and S5.

Our results showed that accumulation of heat shock proteins (HSPs) was strongly affected by drought. Proteins belonging to family of small HSP (sHSP) increased their accumulations in roots of both tested barley genotypes (**Figure 4**; Supplementary Tables S2 and S3). Many of the identified HSP70 isoforms diminished their abundance in both varieties; however, constitutive accumulation levels of these proteins were higher in Cam/B1/CI than in Maresi (**Figures 4** and **5**; Supplementary Tables S2–S5). Members of HSP100 family, caseino-lytic protease (ClpP) and its associated chaperone isoforms (ClpC), decreased their accumulation in Maresi leaves during the response to water deficit. Opposite, in Cam/B1/CI leaves ClpP accumulated to higher levels with drought (**Figure 5**). In addition, an increase in the abundance of cold-regulated protein (COR), which is typically associated with tolerance to low temperatures (Hajela et al., 1990) was observed in roots of both varieties (**Figure 4**).

### Photosynthesis and Glycolysis

Our proteomic analysis revealed drought induced changes in concentration of proteins constituting the photosystem I (PSI) and PSII reaction centers and energy transfer. Two isoforms of the oxygen-evolving enhancer protein decreased in both cultivars, while accumulation of yet another isoform increased in Maresi (**Figure 5**). The levels of ferredoxin-NADP+ oxidoreductase were diminished in Maresi, whereas cytochrome b6-f exhibited increased accumulation upon drought treatment (**Figure 5**).

We observed drought-induced decrease in the levels of ribulose-1,5-bisphosphate carboxylase/oxygenase (RuBisCO) large subunit in Maresi, whereas in Cam/B1/CI such change was not detected. In both tested genotypes RuBisCO activase B exhibited strong drought-induced accumulation, whereas the level of isoform A was decreased in Maresi. At the same time accumulation of RuBisCO large subunit binding protein declined in both cultivars, especially in Maresi (**Figure 5**).

An isoform of fructose-bisphosphate aldolase that could be involved in Calvin cycle and glycolysis decreased its abundance in drought-stressed Maresi leaves. At the same time the level of another isoform of this enzyme increased in both varieties (**Figure 5**). Accumulation of enolase, which is a penultimate enzyme in glycolysis was reduced in Maresi, but increased in Cam/B1/CI leaves. Similarly, the abundance of chloroplastic glyceraldehyde 3-phosphate dehydrogenase declined in Maresi, while another isoform increased its concentration in Cam/B1/CI leaves (**Figure 5**). Decrease in fructokinase accumulation level was observed in root tissue of both tested varieties; however, in Cam/B1/CI one isoform of this enzyme accumulated to higher extent (**Figure 4**). Observed changes suggest that glycolysis is compromised in Maresi, but maintained or even elevated in Cam/B1/CI during the response to drought.

**FIGURE 6:**
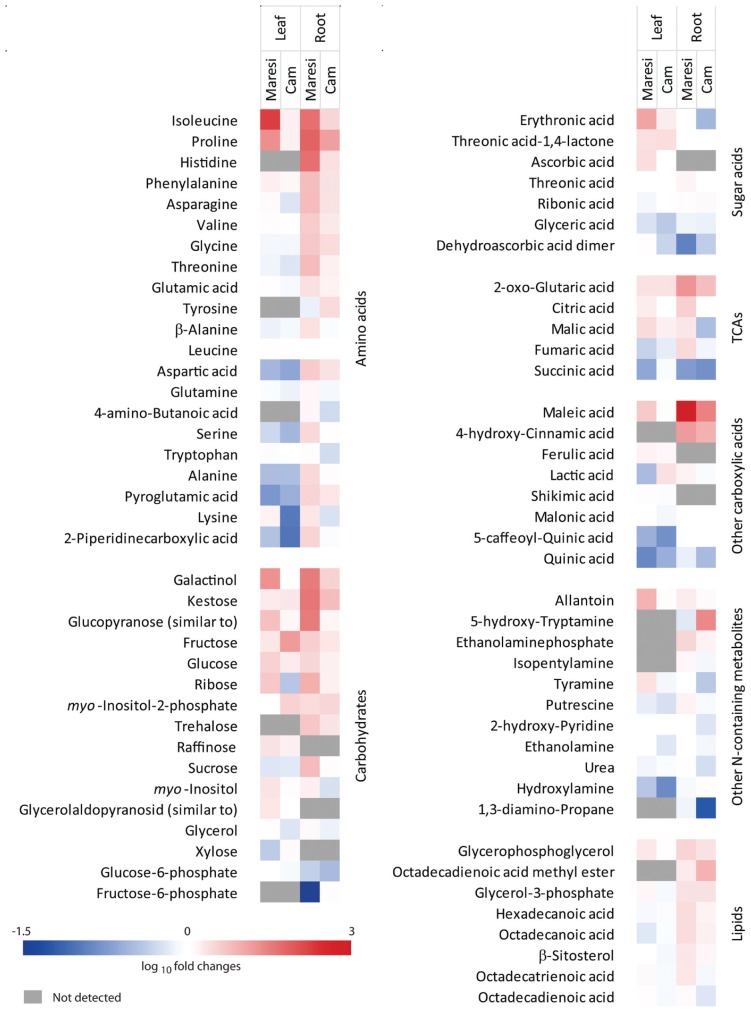
**Drought-induced changes in the accumulation levels of metabolites identified in both tested barley lines.** Quantitative results and statistical significance are shown in Supplementary Table S1.

Mitochondrial pyruvate dehydrogenase E1 subunit alpha (PDHA1) and NADP dependent malic enzyme (NADP-ME), which are involved in redox reactions in glycolysis and Krebs cycle (Tovar-Méndez et al., 2003; Wheeler, 2005), increased their abundances in roots of Maresi and both cultivars, respectively. Observed changes in PDHA1 and NADP-ME accumulation may suggest enhanced activity of Krebs cycle under drought conditions. In accordance with this suggestion, we observed elevated levels of α-ketoglutaric acid in drought stressed leaves and roots of both tested barley lines (**Figure 6**). Contrary, accumulation of succinic acid decreased with drought. Fumaric and citric acids differentiated roots of tested barley lines exhibiting clear up-regulation in Maresi. From other carboxylic acids the most striking drought-induced changes were observed for maleic acid, which increased its accumulation in roots of both tested lines, but only in Maresi leaves. Overall, Maresi revealed higher increases in carboxylic acid accumulation than Cam/B1/CI (**Figure 6**).

### Reactive Oxygen Species

Proteins implicated in ROS generation: hydroxy acid oxidase (HAO, also known as glycolate oxidase) and oxalate oxidase (OXO) exhibited increased accumulation in roots of Maresi and Cam/B1/CI, respectively (**Figure 4**). On the other hand, the putative drought-induced increase in ROS generation was compensated with changes in accumulation of several proteins that may aid plants in circumventing oxidative damage, including enzymes associated with the ascorbate–glutathione cycle. The increased level of ascorbate peroxidase (APX) was observed in leaves of both tested barley lines, but glutathione reductase, which catalyzes the last step in the cycle was up-regulated only in Cam/B1/CI leaves (**Figure 5**). In addition, the accumulation levels of glutathione *S*-transferase (GST), which is involved in glutathione mediated ROS scavenging (Noctor et al., 2012), and 2-cystein peroxiredoxin (2-CP) increased in both lines in response to water deficit (**Figure 5**). Overall, the majority of identified leaf proteins involved in ROS scavenging accumulated to higher extent in both genotypes under water deficiency conditions. In root tissue, the abundance of glyoxalase I/lactoylglutathione lyase was increased in both lines (**Figure 4**). Interestingly, opposite to leaves, other proteins involved in ROS scavenging did not change or even reduced their accumulation levels in roots of both tested lines subjected to drought. This concerns GSTs, APXs, and superoxide dismutases.

A few non-enzymatic components of ROS scavenging system were also identified. Ascorbic acid, which is a principal ROS scavenger in plant cells (Asada, 1992), was detected only in leaves, and its concentration significantly increased after drought treatment in Maresi (**Figure 6**). Similarly, accumulation of *myo*-inositol, which belongs to another group of ROS scavengers (Smirnoff and Cumbes, 1989), was found to be up-regulated exclusively in Maresi leaves. At the same time accumulation of fructose-6-phosphate, which also possess antioxidant properties (Spasojević et al., 2009), decreased in roots of this barley line (**Figure 6**). Also some of the identified sugars were proposed to contribute to ROS scavenging during plant response to drought (Foyer and Shigeoka, 2011). This concerns for instance galactinol (Nishizawa et al., 2008), which revealed the most striking increase in concentration among all detected sugars in roots of both tested genotypes, but only in Maresi leaves (**Figure 6**).

### Osmolytes

Sugars may also play a role in osmotic adjustment. Our analysis revealed that accumulation of several carbohydrates was affected in tissues of both genotypes subjected to drought. Sucrose was the most abundant metabolite identified in studied samples. Despite its high constitutive concentrations, accumulation of this metabolite significantly increased in roots of drought-stressed Maresi plants. Also fructose, ribose and 1-kestose increased their concentrations stronger in Maresi than in Cam/B1/CI roots (**Figure 6**).

Glycine betaine (GB) and proline are two main osmolytes that accumulate in response to various abiotic stresses (Ashraf and Foolad, 2007). The accumulation of betaine aldehyde dehydrogenase (BADH), crucial enzyme involved in GB synthesis, was elevated in roots of both cultivars, but only in Cam/B1/CI leaves (**Figure 5**; Supplementary Tables S2 and S3). However, we did not detect GB during our GC/MS analysis.

The key enzyme involved in proline synthesis, delta-1-pyrroline-5-carboxylate synthetase (P5CS) did not exhibit significant changes in its accumulation pattern in leaves of Cam/B1/CI; however, in Maresi the level of this enzyme was clearly reduced (**Figure 5**). The abundance of an enzyme involved in catabolism of proline, pyrroline-5-carboxylate dehydrogenase, was significantly increased in Cam/B1/CI roots (Supplementary Table S3). Opposite to the changes in P5CS accumulation, metabolomic analyses showed increased proline levels in leaves of both tested cultivars subjected to drought. In Maresi we observed more than 20-fold increase in accumulation of this amino acid, but only 1.8-fold change was registered for Cam/B1/CI leaves (**Figure 6**). Similar pattern was observed in roots; proline levels increased more than 70-fold in Maresi and 15-fold in Cam/B1/CI (Supplementary Table S1). However, this higher fold of change in the concentration of proline in German cultivar was mainly due to clearly lower constitutive content of this amino acid in comparison to Cam/B1/CI plants grown in optimal conditions.

### Amino Acids and Nitrogen Metabolism

Apart of proline, several other amino acids were clearly induced in Maresi, but not in Cam/B1/CI roots. Opposite to roots, in leaves the response of both lines was more similar with predominant reduction in the amino acid cocnentrations. Changes in accumulation of enzymes involved in amino acid biosynthesis also discriminated tested genotypes. In Maresi leaves their accumulation was reduced, whereas in Cam/B1/CI it remained mainly unchanged. Additionally, two enzymes involved in nitrogen flow that use glutamate as a substrate, glutamate dehydrogenase and glutamine synthetase, exhibited increased abundances exclusively in Cam/B1/CI leaves (**Figure 5**). Nitrogen-containing compounds, putrescine and hydroxylamine, significantly decreased their levels in leaves of both genotypes subjected to drought (**Figure 6**). In contrast, the level of allantoin, which is a nitrogen-transporting compound (Watanabe et al., 2014), significantly increased, but only in Maresi leaves. Interestingly, this particular metabolite has been reported as an important player in the abscisic acid-mediated abiotic stress tolerance (Watanabe et al., 2014).

### Secondary Metabolism

We observed a clear decrease in the levels of the phenylalanine ammonia-lyase (PAL), which is an important regulatory point between primary and secondary metabolism (Vogt, 2010), in Cam/B1/CI roots and leaves (**Figures 4** and **5**). However, accumulation of PAL substrate phenylalanine in the Syrian genotype did not reveal statistically significant changes in response to drought (**Figure 6**). During our GC/MS analysis we identified only a limited number of phenylpropanoid-type products including *p*-coumaric acid in roots as well as ferulic and chlorogenic acid in leaves. However, only the chlorogenic acid content decreased in both analyzed genotypes (**Figure 6**).

## Discussion

### Differential Adaptation to Drought of Both Tested Barley Lines

Our phenotypic analysis revealed a significant drought-related reduction of plant yield for Maresi, but not for Cam/B1/CI (**Table 1**). Other phenotypic traits and RWC were also more affected by drought in Maresi. The worse adaptation of this cultivar to water deficiency could be additionally inferred from the observed negative effects of drought on proteins involved in carbon assimilation and catabolism. For instance, we found a significant reduction in the abundance of RuBisCO large subunit and RuBisCO large subunit binding protein in Maresi, but not Cam/B1/CI leaves (**Figure 5**). Similarly, the recent proteomic studies revealed decrease in the accumulation levels of RuBisCO large subunit, RuBisCO activases and RuBisCO large subunit binding protein in drought-sensitive barley genotypes (Ashoub et al., 2013; Kausar et al., 2013). In addition to RuBisCO, also enzymes involved in nitrogen assimilation and amino acid biosynthesis were more negatively affected by drought in Maresi than in Cam/B1/CI. The level of leaf nitrogen plays an important role in photosynthetic acclimation of the plant to environmental variables (Ainsworth et al., 2003). It was shown in several studies that leaf nitrogen decreases gradually as the drought progresses and this can be related to photosynthetic apparatus damage (Sinclair et al., 2000; Xu and Zhou, 2006).

The enhanced susceptibility of Maresi plants to water deficit seems to correspond with proteomic and metabolomic responses in leaves of this genotype, where higher number of proteins and metabolites was influenced by drought as compared with Cam/B1/CI. However, in roots, the situation was opposite – more proteins and metabolites changed their accumulation pattern in Cam/B1/CI. It is possible that the rapid and pronounced induction of defensive mechanisms in roots contribute to the enhanced tolerance of this line to water deficit. Conversely, some of the changes in the protein and metabolite accumulation observed in Maresi leaves may be linked with negative consequences of drought rather than with drought-protective mechanisms.

### Molecular Chaperons May Help in Drought Tolerance

Many of the proteins identified during this study are known as general abiotic stress indicators. These include several of HSPs, which constitute a protein family ubiquitous among prokaryotes and eukaryotes. For instance, we found constitutive accumulation levels of HSP70s higher in Cam/B1/CI than in Maresi (Supplementary Tables S2–S5). Similarly, Wendelboe-Nelson and Morris (2012) reported higher constitutive HSP70 levels in the drought-tolerant compared with the susceptible barley genotype. In addition, accumulation of one of the HSP70 leaf isoforms increased with drought in Cam/B1/CI, but decreased in Maresi (**Figure 5**). Kausar et al. (2013) also reported increase of HSP70 accumulation in drought-tolerant barley genotype, whereas in the drought-sensitive genotype the decline was observed. This indicates that in barley constitutive levels of HSP70 as well as changes in the accumulation of this chaperone could correlate with drought resistance. Similarly, to the mentioned above HSP70 isoform, we observed drought induced decrease in ClpP accumulation in Maresi leaves, while at the same time levels of this protein increased in Cam/B1/CI (**Figure 5**). Rosano et al. (2011) revealed a molecular chaperone function of Clp/HSP100 from *Arabidopsis thaliana* and showed its contribution to protein import into the chloroplast. In this species, the regulatory domain of chloroplast targeted Clp complex, encoded by the nuclear gene *Early Responsive to Dehydration 1*, is induced by water stress in ABA-independent manner (Nakashima et al., 1997). Up-regulation of Clp proteins observed in wheat seedlings subjected to water deficiency was proposed to be linked with drought-induced changes in photosynthesis and RuBisCO content (Demirevska et al., 2008).

It is known that the refolding of non-native proteins by ATP-dependent chaperones, including HSP70s is facilitated by the activity of sHSPs (Lee and Vierling, 2000). We found that the sHSP 16.9 isoforms increased their accumulation in roots of both tested lines during the response to drought (**Figure 4**). Similarly, Guo et al. (2009) reported that a gene encoding the sHSP family member was induced under drought stress in tolerant and sensitive barley genotypes. Overall, these findings suggest that changes in the accumulation levels of HSP70 and Clp/HSP100 may contribute to the enhanced drought tolerance of the Cam/B1/CI line. Opposite to these two HSP subclasses, members of the sHSP subfamily respond to water deficit, but are rather not among the traits that determine the difference in drought resistance observed between the two tested barley lines.

### ROS Production and Scavenging Contributes to the Difference in Drought Resistance between Cam/B1/CI and Maresi

Our proteomic analysis revealed drought induced changes in HAO and OXO accumulation (**Figure 4**). These two enzymes are involved in H_2_O_2_ production in peroxisomes and apoplast respectively (Mittler et al., 2004). Similar to our results, Kausar et al. (2013) reported increased accumulation of OXO under drought stress only in a drought-tolerant barley genotype indicating that enhanced production of H_2_O_2_ is not necessarily disadvantageous for plant fitness during the response to drought. Plants possess a number of mechanisms facilitating efficient neutralization of free radicals, including water/water cycle in chloroplasts, ascorbate–glutathione cycle and reactions related to the activity of peroxidases and catalases (Mittler, 2002). The observed increase in APX accumulation in leaves of Maresi and even stronger in Cam/B1/CI combined with elevated levels of glutathione reductase in Cam/B1/CI indicates increased activity of ascorbate–glutathione cycle pathway during drought, particularly in the more resistant Syrian genotype. Similarly to our findings, Wendelboe-Nelson and Morris (2012) also reported more pronounced increase in APX accumulation in the drought-tolerant barley line. Opposite to the changes in APX levels, ascorbic acid accumulation increased in Maresi, but not in the Cam/B1/CI (**Figure 6**). However, leaves of the Syrian line accumulated constitutively higher amounts of ascorbic acid as compared with Maresi (Supplementary Image S5), suggesting that elevated levels of this metabolite in naïve plants could be linked with adaptation to dry climate. Other enzymes linked with the ascorbate–glutathione cycle, which increased their accumulation during drought included a GST and a glyoxalase (**Figure 4**). However, from those two only GST was up-regulated in Cam/B1/CI, but not Maresi (**Figure 4**). The GST activity can be mediated by H_2_O_2_ and overexpression of a GST from *Pyrus pyrifolia* in tobacco increased substantially available amount of enzymatic and non-enzymatic components of ascorbate–glutathione cycle suggesting positive contribution of GSTs to abiotic stress tolerance (Liu et al., 2013). Similarly to our results other studies on barley indicated that GST accumulation during the response to drought only in drought tolerant, but not in sensitive genotypes (Guo et al., 2009; Kausar et al., 2013).

Apart from glutathione and ascorbic acid also other small molecules may contribute to ROS detoxification. For instance, Spasojević et al. (2009) showed that fructose-phosphates have ROS scavenging properties that are more pronounced from those of fructose or glucose. This suggested that fructokinases may play a role ROS scavenging under drought stress (Fulda et al., 2011). In this context it seems of importance that that the ratio of fructose-phosphate to fructose was clearly decreased in drought stressed Maresi, but not Cam/B1/CI roots. These changes correlated with the observation that one out of two identified fructokinases was reduced its accumulation in roots of Maresi, but not of Cam/B1/CI (**Figure 4**).

Overall, our data indicated that water deficit affects several key players of redox balance in barley. Among those we found APX and glutathione reductase as the components whose response to drought differentiated between Maresi and Cam/B1/CI and therefore could be considered to contribute to the enhanced drought tolerance of the Syrian genotype. In addition higher constitutive ascorbic acid accumulation and the maintained during drought fructose-phosphate to fructose ratio could be of significance for the response to water deficit.

### Constitutive Accumulation of Osmoprotective Metabolites Enhances Drought Resistance

Several proteins and metabolites identified in this study are related to osmotic processes, which are severely affected by drought. For instance, elevated proline levels are supposed to enhance cell tolerance toward different kinds of osmotic stresses (Zhu et al., 1998). We observed substantial differences in accumulation of enzymes involved in proline biosynthesis and catabolism (5PCS and P5CDH), as well as clear differences in abundance of this amino acid between Maresi and Cam/B1/CI. Proline accumulation increased significantly during the response to drought in Maresi leaves and roots (**Figure 6**). Interestingly, the synthesis of amino acids with osmoprotective properties (including proline) could be linked with the reallocation of nitrogen derived from RuBisCO degradation (Aranjuelo et al., 2011). This correlated with the elevated decrease in the accumulation of RuBisCO-related proteins that we observed in Maresi, as compared with Cam/B1/CI (**Figure 6**). Opposite to the changes in proline accumulation, at the same time 5PCS levels decreased in leaves of this variety (**Figure 5**). As high proline amounts may trigger cell damage (Hellmann et al., 2000), observed in Maresi drop of P5CS accumulation may indicate a feedback regulation initiated by the huge drought-induced increase in proline concentration. Similar to glutathione, constitutive accumulation level of proline in Cam/B1/CI leaves was already relatively high and increased only moderately upon drought, which correlated with lack of changes in P5CS accumulation (**Figures 5** and **6**; Supplementary Image S5). Interestingly, wheat seedlings with altered tolerance to water deficit did not differ regarding proline accumulation. However, the resistant variety was able to accumulate and metabolize this compound more rapidly (Nayyar and Walia, 2003). Thus, it seems that the key aspect of drought tolerance is not only proline accumulation level, but also appropriate regulation of the biosynthesis and metabolism of this amino acid. In addition to proline, we also observed significant differences in the accumulation levels of potentially osmoprotective disaccharides and trisaccharides in the control and stressed plants. Cam/B1/CI was the genotype with a substantial difference in 1-kestose concentration between control and drought conditions (**Figure 6**). In addition, several monosaccharides accumulated constitutively to higher levels in Cam/B1/CI as compared with Maresi (Supplementary Image S5). It seems possible that the elevated sugar levels contribute to the better osmotic protection in the Syrian line to tolerate osmotic stress.

### A Link between Phenylpropanaoid Pathway and Drought Tolerance

We found PAL among the enzymes whose accumulation was consistently down-regulated in Cam/B1/CI but not in Maresi (**Figures 4** and **5**). Initiated with this enzyme phenylpropanoid pathway is crucial for the biosynthesis of different products which are critical in plant development and plant adaptation to the environment (Dixon and Paiva, 1995). These metabolites include lignin, phenolic acids, and flavonoids. Interestingly, accumulation of caffeoyl-CoA *O*-methyltransferase, which is involved in the phenolic acid/lignin branch of phenylpropanoid metabolism (Boerjan et al., 2003), also decreased in drought stressed Cam/B1/CI roots (**Figure 4**). A proteomic study on maize suggested that lignin biosynthesis suppression might play a role in β-aminobutyric acid-induced drought resistance (Macarisin et al., 2009). In accordance with this functional correlation, *A. thaliana pal1 pal2* double-knockout mutant, which produces only 30% of the wild-type lignin content, is significantly more resistant to drought than the wild-type plants (Rohde et al., 2004; Huang et al., 2010). Cumulatively, these results support a potential link between reduced lignin biosynthesis/deposition and drought tolerance. However, molecular mechanisms of this link remain obscure.

## Conclusion

Our results indicate that activity of molecular chaperons as well as production of osmoprotectants and antioxidants may collectively mediate enhanced resistance to water deficit observed in Cam/B1/CI line. Interestingly, it seems that the elevated constitutive accumulation of several molecular components of these defense mechanisms, including HSP70, proline, carbohydrates and ascorbic acid, is indispensable for the proper response to water deficit. Additional drought-triggered increase in accumulation of certain proteins and metabolites including HSP70, Clp, APX, GST, and carbohydrates is also of significance. Moreover, down-regulation of secondary metabolism, particularly lignin biosynthesis, may also contribute to the enhanced drought tolerance observed in Cam/B1/CI. However, it should be noted that we observed several clear changes in protein and metabolite accumulation that were similar in both tested lines. At the currant stage it can be not excluded, that although these changes were not discriminating the response of Cam/B1/CI and Maresi, they may still significantly contribute to drought resistance.

## Author Contributions

The experimental set up was designed by KC, PR, BS, AS, MSu, TA, and MSt. KC, PR, BS, ŁM, DC, AK, KM, PO, and KK performed the experiments and acquired the data. KC, PR, BS, AS, and PK analyzed the data. KC, PR, BS, AS, PK, PB, and MSt interpreted the results and wrote the manuscript with input from all other authors. MSt supervised the project.

## Conflict of Interest Statement

The authors declare that the research was conducted in the absence of any commercial or financial relationships that could be construed as a potential conflict of interest.

## Footnotes

1 http://www.faostat.fao.org

2 www.vsni.co.uk
