# Supplementary material for: Analysis of Drought-Induced Proteomic and Metabolomic Changes in Barley (Hordeum vulgare L.) Leaves and Roots Unravels Some Aspects of Biochemical Mechanisms Involved in Drought Tolerance
Source: Front Plant Sci. 2016 Jul 26;7:1108. doi: 10.3389/fpls.2016.01108 (PMC4962459; doi:10.3389/fpls.2016.01108)
Supplement: Supplementary file 3 [file Image_3.PDF]

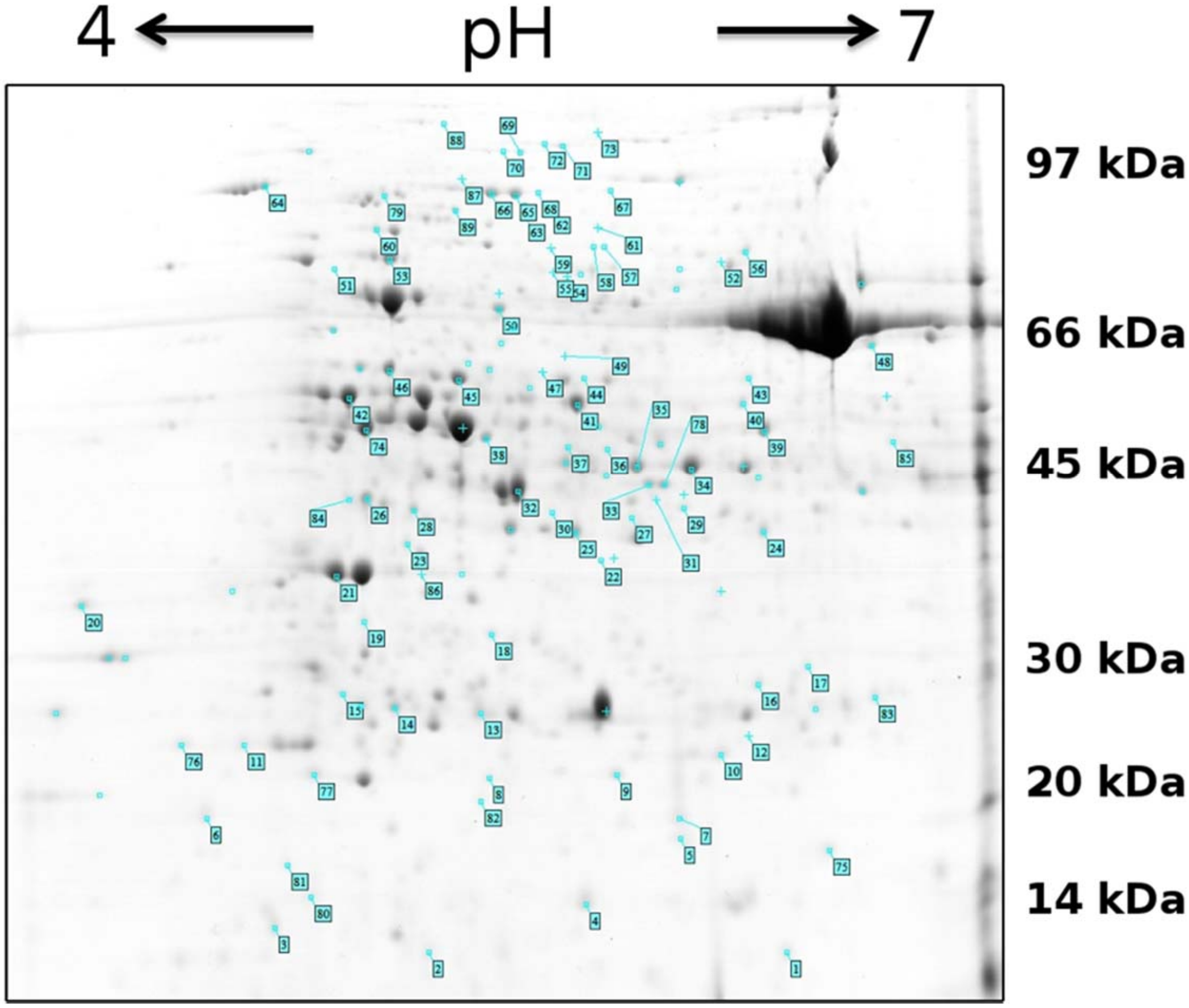

Supplementary Image S3. Representative two-dimensional electrophoretic gel obtained during analysis of protein extracts extracted from Maresi leaves. Protein spot labels according to Supplementary Table S4.
