# Supplementary material for: Analysis of Drought-Induced Proteomic and Metabolomic Changes in Barley (Hordeum vulgare L.) Leaves and Roots Unravels Some Aspects of Biochemical Mechanisms Involved in Drought Tolerance
Source: Front Plant Sci. 2016 Jul 26;7:1108. doi: 10.3389/fpls.2016.01108 (PMC4962459; doi:10.3389/fpls.2016.01108)
Supplement: Supplementary file 6 [file Table_1.PDF]

Supplementary Table S1. Quantitative analysis of metabolite accumulation levels in leaves and roots of control and drought-stressed Maresi and Cam/B1/CI plants. Direction of drought induced changes in metabolite accumulation indicated as: -1: significant down-regulation, 0: no significant difference, 1: significant up-regulation (P < 0.01). Results of quantitative analyses for identified barley metabolites are normalized to an internal standard, averaged over technical replicates and transformed to a log2 scale.

#	Metabolite	Leaf										Root									
		ANOVA tests results (P-value) for			Mean values				Std. error	Direction of change under drought		ANOVA tests results (P-value) for			Mean values				Std. error	Direction of change under drought	
					Maresi		Cam/B1/CI								Maresi		Cam/B1/CI				
		Variety	Drought	Variety x drought interaction	Control	Drought	Control	Drought		Maresi	Cam/B1/CI	Variety	Drought	Variety x drought interaction	Control	Drought	Control	Drought		Maresi	Cam/B1/CI
1	A105001-Lactic acid (2TMS)				15.57	14.19	16.93	18.25	0.57	0	0				15.07	15.77	15.34	15.17	0.15	0	0
2	A105003-Pyridine; 2-hydroxy- (1TMS)				17.02	17.00	16.37	16.38	0.12	0	0				12.82	12.76	13.01	12.42	0.34	0	0
3	A110002-Hydroxylamine (3TMS)		0.000		15.71	14.53	16.31	13.90	0.12	-1	-1				12.78	12.58	12.83	13.06	0.04	0	0
4	A114002-NA114002 (classified unknown)				20.60	21.09	20.14	19.94	0.21	0	0	0.009			18.13	18.77	17.92	17.64	0.09	1	0
5	A115001-Isopentylamine (2TMS)														16.92	17.48	16.84	16.56	0.07	0	0
6	A119001-Phosphoric acid monomethyl ester (2TMS)	0.007	0.000	0.000	16.58	18.90	17.82	18.19	0.04	1	1		0.002		13.66	16.46	13.71	16.08	0.25	1	1
7	A122001-Valine (2TMS)				17.41	17.71	17.04	17.08	0.12	0	0		0.005		15.61	17.72	16.03	17.02	0.20	1	0
8	A122003-Malonic acid (2TMS)				15.63	15.72	15.69	15.41	0.14	0	0										
9	A127002-Urea (2TMS)				14.45	14.14	14.83	14.73	0.11	0	0				13.43	13.64	14.42	13.71	0.38	0	0
10	A128002-Ethanolamine (3TMS)				16.80	16.80	17.25	16.72	0.19	0	0				16.25	16.35	16.65	16.36	0.14	0	0
11	A129001-Phosphoric acid (3TMS)				20.56	19.93	20.30	19.42	0.32	0	0				18.00	18.02	17.92	17.93	0.55	0	0
12	A129002-Leucine (2TMS)				16.27	16.45	16.68	16.65	0.20	0	0				12.08	12.08	14.37	14.33	1.15	0	0
13	A129003-Glycerol (3TMS)				16.58	16.78	17.02	16.44	0.14	0	0				15.69	16.11	16.20	15.85	0.46	0	0
14	A132002-Isoleucine (2TMS)				12.72	20.40	14.96	15.72	2.47	0	0				14.71	20.43	12.82	14.48	2.18	0	0
15	A132003-Proline (2TMS)	0.004	0.000	0.001	17.66	22.21	20.76	21.59	0.18	1	1		0.000		14.54	20.76	16.45	20.35	0.43	1	1
16	A133001-Glycine (3TMS)				19.49	19.20	19.55	19.26	0.13	0	0		0.003		14.27	16.51	14.84	16.32	0.21	1	1
17	A133003-Maleic acid (2TMS)		0.001	0.003	17.72	19.92	19.21	19.48	0.13	1	0		0.000		8.37	18.49	12.97	17.91	0.65	1	1
18	A134001-Succinic acid (2TMS)				18.16	16.37	18.51	18.36	0.35	-1	0		0.009		14.61	12.57	15.41	13.14	0.42	-1	-1
19	A135003-Glyceric acid (3TMS)		0.002		17.87	17.23	18.07	16.97	0.10	-1	-1				9.64	9.31	8.66	8.28	0.93	0	0
20	A137001-Fumaric acid (2TMS)		0.000		16.61	15.65	16.18	15.73	0.06	-1	-1				12.56	14.12	13.47	13.16	0.56	1	0
21	A137004-2-Piperidinecarboxylic acid (2TMS)				17.88	16.64	16.87	14.02	0.43	0	-1				13.84	15.63	14.33	14.21	0.41	1	0
22	A138001-Serine (3TMS)	0.002	0.001		19.93	19.11	19.31	17.84	0.11	-1	-1				16.16	17.77	16.64	16.84	0.46	1	0
23	A138002-Alanine (3TMS)	0.009	0.002		18.23	16.87	17.30	15.97	0.15	-1	-1				14.09	15.69	14.50	14.81	0.44	1	0
24	A140001-Threonine (3TMS)				18.33	18.02	18.49	17.94	0.08	0	-1				13.46	16.21	14.92	15.73	0.42	1	0
25	A140005-Threonic acid-1;4-lactone (2TMS)				13.93	15.25	12.87	14.24	0.62	0	0										
26	A144001-Alanine; beta- (3TMS)				15.05	14.69	14.41	14.15	0.12	0	0				12.95	14.25	13.84	13.76	0.11	1	0
27	A144004-NA				18.23	18.24	17.97	17.75	0.26	0	0				13.42	13.69	13.58	12.96	0.10	0	-1
28	A145015-NA145015														13.91	14.33	13.98	13.56	0.11	0	0
29	A148003-NA	0.000	0.000	0.000	13.73	7.66	7.66	7.66	0.02	-1	0	0.006	0.008		10.58	12.12	9.03	10.45	0.16	1	1
30	A149001-Malic acid (3TMS)				21.58	23.12	21.94	22.70	0.31	1	0				16.85	17.85	18.85	17.52	0.72	0	0
31	A152002-Aspartic acid (3TMS)		0.000		20.03	18.57	20.05	18.24	0.08	-1	-1		0.003		15.36	17.43	16.10	17.27	0.10	1	1
32	A153002-Pyroglutamic acid (2TMS)		0.001		19.74	17.61	19.78	18.12	0.18	-1	-1				18.62	20.37	18.84	19.93	0.36	0	0
33	A153003-Butanoic acid; 4-amino- (3TMS)														17.15	17.71	18.00	17.23	0.41	0	0
34	A154001-Erythronic acid (4TMS)	0.004			13.12	16.80	17.57	18.41	0.41	1	0	0.000			10.02	10.02	15.21	13.71	0.34	0	-1
35	A156001-Threonic acid (4TMS)				10.93	10.93	10.93	10.93	0.00	0	0	0.000			11.18	11.81	8.51	8.51	0.12	0	0
36	A158004-Glutaric acid; 2-oxo- (1MEOX) (2TMS) MP	0.001	0.001		15.22	16.48	16.42	17.67	0.11	1	1		0.000		11.13	15.47	11.54	14.14	0.25	1	1
37	A159003-NA	0.002	0.000		14.51	16.46	15.47	17.00	0.08	1	1										
38	A163001-Glutamic acid (3TMS)				20.07	20.29	20.99	20.77	0.23	0	0		0.001		16.78	18.09	17.33	18.09	0.13	1	1
39	A164001-Phenylalanine (2TMS)				16.54	17.30	14.86	15.40	0.69	0	0				14.68	17.28	15.45	16.66	0.55	1	0
40	A164007-Asparagine (4TMS) BP1				17.13	17.51	17.99	17.40	0.23	0	0				16.08	18.76	16.69	17.94	0.66	1	0
41	A164010-Propane; 1;3-diamino- (4TMS)														10.33	10.01	10.63	7.13	3.35	0	0
42	A165001-Xylose (1MEOX) (4TMS) MP				13.82	12.77	13.69	14.09	0.30	0	0										
43	A168002-Ribose (1MEOX) (4TMS) MP				12.01	14.29	12.71	11.57	1.24	0	0				14.61	17.84	15.80	16.59	0.43	1	0
44	A174008-Glutamine (4TMS)	0.008			17.34	17.24	19.45	19.10	0.25	0	0				18.64	19.13	18.73	18.49	0.22	0	0
45	A175002-Putrescine (4TMS)		0.002		15.18	14.74	15.60	14.92	0.07	-1	-1				15.80	16.50	15.96	15.76	0.15	0	0
46	A177001-Ribonic acid (5TMS)	0.004			15.93	15.65	17.23	17.20	0.22	0	0				12.34	12.58	13.02	13.42	0.51	0	0
47	A177002-Glycerol-3-phosphate (4TMS)				15.01	15.54	15.62	15.40	0.13	0	0				9.33	10.52	10.72	11.93	0.85	0	0
48	A177014-Ethanolaminephosphate (4TMS)														14.22	15.90	14.41	15.09	0.27	0	0
49	A178003-NA				16.64	15.57	17.97	17.21	0.35	0	0				15.55	15.17	14.64	15.02	0.87	0	0
50	A179010-NA				15.68	15.50	15.84	15.04	0.13	0	-1				13.18	9.47	11.88	11.39	1.70	0	0
51	A181002-Shikimic acid (4TMS)				17.74	18.05	17.83	17.70	0.10	0	0										
52	A182004-Citric acid (4TMS)	0.009	0.003	0.004	20.44	21.32	20.52	20.56	0.06	1	0				16.05	18.00	17.16	17.10	0.49	1	0
53	A185001-Quinic acid (5TMS)				18.74	16.29	19.49	17.78	0.70	0	0				13.24	12.85	14.20	12.81	0.35	0	0
54	A185002-Dehydroascorbic acid dimer (2MEOX) MP				18.06	18.23	16.92	16.00	0.34	0	0				7.95	5.36	4.80	3.80	0.60	0	0
55	A187002-Fructose (1MEOX) (5TMS) MP	0.001	0.004		16.85	17.90	19.55	23.66	0.37	0	1	0.007			18.87	20.87	21.22	22.33	0.16	1	0
56	A189002-Glucose (1MEOX) (5TMS) MP				20.65	22.53	22.46	23.40	0.31	1	0				18.69	20.35	19.97	20.78	0.30	1	0
57	A189007-Allantoin (4TMS)				15.32	18.44	18.66	18.95	0.55	1	0				16.93	17.84	16.86	17.26	0.14	0	0
58	A191004-Tyramine (3TMS)				12.19	13.48	12.56	12.27	0.40	0	0				15.03	15.00	15.46	14.34	0.63	0	0
59	A192003-Lysine (4TMS)				13.17	13.87	13.07	10.33	0.50	0	-1				14.93	15.98	15.44	14.81	0.57	0	0
60	A194002-Tyrosine (3TMS)														12.56	12.17	10.08	11.61	0.21	0	0
61	A195001-Cinnamic acid; 4-hydroxy-; trans- (2TMS)														8.63	12.65	8.20	11.41	1.67	0	0
62	A195002-Ascorbic acid (4TMS)	0.002			14.97	16.35	17.88	17.89	0.24	1	0										

63 A197003-similar to Glucopyranose (5TMS)	0.008	0.004		17.05	19.62	19.32	19.86	0.24	1	0				13.17	18.45	16.87	17.48	1.19	1	0
64 A197007-NA197007				17.50	19.08	18.71	19.33	0.24	1	0				12.97	16.99	15.69	16.20	1.05	1	0
65 A199003-NA199003 (classified unknown)														5.13	5.13	8.16	8.19	1.52	0	0
66 A203003-NA				14.52	12.59	16.23	17.05	0.66	0	0				11.07	13.91	12.52	14.19	0.72	1	0
67 A205001-Hexadecanoic acid (1TMS)				17.96	17.75	18.26	18.13	0.10	0	0				14.64	16.03	15.42	16.11	0.31	1	0
68 A209002-Inositol; myo- (6TMS)				17.98	19.13	17.99	18.26	0.17	1	0				15.17	15.96	16.26	15.60	0.26	0	0
69 A210001-Ferulic acid; trans- (2TMS)				12.90	13.65	13.91	14.48	0.17	0	0										
70 A210010-Octadecadienoic acid methyl ester; 9;12-(Z;Z)-; n-														7.36	8.28	7.36	10.49	0.00	0	1
71 A211001-NA211001				16.54	17.68	17.61	18.29	0.21	1	0				9.08	7.92	7.92	7.92	0.58	0	0
72 A215003-Histidine (4TMS)														3.48	9.21	3.28	4.61	0.76	1	0
73 A217007-NA	0.004			13.39	15.12	16.38	17.16	0.33	1	0										
74 A218002-Glycerophosphoglycerol (5TMS)				13.62	14.58	13.76	13.99	0.26	0	0		0.001		11.52	13.30	12.09	13.25	0.10	1	1
75 A221003-Octadecadienoic acid; 9;12-(Z;Z)- (1TMS)				17.04	17.26	17.38	17.18	0.06	0	0				14.64	15.00	15.07	14.49	0.35	0	0
76 A222003-Octadecatrienoic acid; 9;12;15-(Z;Z;Z)-; n- (1TMS)				16.83	17.23	17.41	17.23	0.07	1	0				12.69	13.74	13.81	13.58	0.46	0	0
77 A223001-Tryptophan (3TMS)				17.83	17.70	18.17	18.14	0.09	0	0				16.16	16.48	16.31	15.56	0.25	0	0
78 A225002-Octadecanoic acid (1TMS)				16.56	16.03	16.70	16.60	0.15	0	0				12.82	14.25	13.11	13.92	0.40	1	0
79 A228001-NA				10.17	11.30	10.59	11.41	0.30	0	0				10.85	10.88	11.56	11.32	0.49	0	0
80 A231002-similar to Glycerolaldopyranosid (6TMS)	0.002			14.69	15.78	16.61	16.77	0.17	1	0										
81 A232002-Fructose-6-phosphate (1MEOX) (6TMS) MP														9.28	5.09	8.98	9.23	0.63	-1	0
82 A233002-Glucose-6-phosphate (1MEOX) (6TMS) MP				14.12	14.22	15.30	15.10	0.28	0	0				12.73	11.76	12.95	11.54	0.37	0	0
83 A236005-NA		0.001		15.19	15.94	15.50	15.82	0.04	1	1				10.88	12.25	11.42	12.90	0.03	0	1
84 A237001-NA				9.93	10.52	12.63	14.83	0.60	0	0				11.51	12.85	12.89	13.28	0.20	1	0
85 A243003-Inositol-2-phosphate; myo- (7TMS)				5.43	5.43	5.43	7.26	0.46	0	0				9.98	11.46	9.87	11.58	0.66	0	0
86 A248001-Tryptamine; 5-hydroxy- (4TMS)														4.82	4.32	7.88	12.59	2.03	0	0
87 A250001-NA	0.000			14.35	15.29	18.20	18.36	0.15	1	0										
88 A254002-NA				13.06	15.99	14.29	16.23	0.54	1	0										
89 A264001-Sucrose (8TMS)				26.60	26.03	25.91	25.41	0.14	0	0				22.40	25.13	24.38	24.63	0.67	1	0
90 A270003-NA														7.15	8.08	7.51	9.72	1.86	0	0
91 A274002-Trehalose; alpha;alpha'-; D- (8TMS)														13.94	16.25	15.02	16.14	0.12	0	0
92 A281001-NA	0.000	0.000	0.000	2.19	14.47	13.23	13.41	0.22	1	0										
93 A296003-NA296003 (classified unknown)		0.000		15.64	13.71	15.46	13.51	0.14	-1	-1										
94 A299002-Galactinol (9TMS)	0.000	0.000	0.000	10.70	15.15	10.70	10.70	0.04	1	0	0.000	0.000	0.000	10.09	15.36	10.09	11.91	0.00	1	1
95 A300001-NA				15.77	10.68	16.23	15.53	0.47	-1	0	0.004	0.001	0.008	13.70	10.02	14.02	12.96	0.29	-1	-1
96 A311002-NA		0.000	0.004	15.36	16.60	15.80	16.31	0.06	1	1				11.97	13.68	13.19	13.05	0.47	1	0
97 A319001-Quinic acid; 5-caffeoyl-; trans- (6TMS)		0.001		15.37	13.73	15.16	12.89	0.22	-1	-1										
98 A338002-Sitosterol; beta- (1TMS)				14.93	14.97	15.08	14.83	0.08	0	0				13.85	14.88	14.17	14.66	0.38	0	0
