# Supplementary material for: Analysis of Drought-Induced Proteomic and Metabolomic Changes in Barley (Hordeum vulgare L.) Leaves and Roots Unravels Some Aspects of Biochemical Mechanisms Involved in Drought Tolerance
Source: Front Plant Sci. 2016 Jul 26;7:1108. doi: 10.3389/fpls.2016.01108 (PMC4962459; doi:10.3389/fpls.2016.01108)
Supplement: Supplementary file 7 [file Table_2.PDF]

Table S2. List of drought-responsive proteins in Maresi root extracts.

Spot no.	Protein name	Organism	ANOVA	MARESI control	Maresi stress	Stress/Control	Accumulation level	Method of identification	Score	% coverage	pI (theoretical)	Mw (theoretical)	pI (experimental)	Mw (experimental)	Accession number	Position on the gel
1	Unidentified		0,010367	0,182689	0,308785	1,690222181	INCREASED						4.59	13232		
2	Unidentified		0,00383597	0,0507191	0,186662	3,680309785	INCREASED						6.30	14335		
3	Heat shock 16.9 kDa protein	*Triticum aestivum*	1,46E-05	0,0473651	0,487252	10,28715235	INCREASED	MS/MS	106	15	5.80	16868	5.79	15498	HS16A_WHEAT	A13
4	Unidentified	*Hordeum vulgare*	7,85E-06	0,0275506	0,403508	14,64606941	INCREASED						4.46	16395		
5	Heat shock 16.9 kDa protein	*Arabidopsis thaliana*	0,00531114	0,0395486	0,152858	3,865067284	INCREASED	MS/MS	148	15	5.36	17593	5.37	16754	HS17C_ARATH	A12
6	Heat shock 16.9 kDa protein	*Triticum aestivum*	8,90E-08	0,0528721	0,414164	7,833318518	INCREASED	MS/MS	92	11	5.83	16868	5.48	16718	HS16A_WHEAT	
7	Cold-regulated protein	*Hordeum vulgare*	2,39E-06	0,0509343	0,370538	7,274822664	INCREASED	PMF	120	79	4.93	17659	4.82	16863	Q9FSI8_HORVU	A10
8	Hydroxyacid oxidase 1	*Zea mays*	0,00261121	0,0394404	0,0874908	2,218304074	INCREASED	PMF	68	24	8.72	40424	6.74	17610	B6UCS5_MAIZE	
9	Translation initiation factor 5A	*Triticum aestivum*	0,00161269	0,162425	0,283741	1,746904725	INCREASED	PMF	67	45	5.76	17599	6.14	19624	Q3S4H9_WHEAT	
10	NADPH-dependent FMN reductase	*Hordeum vulgare*	0,00441695	0,0241285	0,0628055	2,602959156	INCREASED	PMF	78	45	6.28	18829	6.58	21032	gi|326508766	
11	Universal stress protein A-like protein-like isoform 1	*Brachypodium distachyon*	0,00272864	0,0210632	0,0466062	2,212683733	INCREASED	PMF	86	29	5.78	20173	6.36	23185	I1ID24_BRADI	
12	Unidentified		8,84E-04	0,0447173	0,0992325	2,219107594	INCREASED						5.08	24795		
13	Unidentified		0,00467712	0,0957465	0,234123	2,445238207	INCREASED						6.54	27333		
14	Germin-like protein 8-5	*Oryza sativa*	7,77E-06	0,302375	0,142958	0,472783795	decreased	MS/MS	120	7	6.40	24400	6.73	28666	GL85_ORYSJ	
15	Ribosomal protein S1-like RNA-binding domain/zinc knucle	*Hordeum vulgare*	0,00872068	0,0466946	0,0943998	2,02164276	INCREASED	PMF	158	65	5.95	25238	6.29	33770	gi|326502466	
16	P-loop containing nucleoside triphosphate hydrolase	*Hordeum vulgare*	1,30E-05	0,0319094	0,163374	5,119933311	INCREASED	PMF	114	40	7.08	38699	6.08	36096	gi|326522022	A14
17	14-3-3-like protein GF14-C	*Oryza sativa*	1,59E-04	0,0567486	0,107642	1,896822124	INCREASED	MS/MS	67	6	4.78	28979	4.71	36742	14333_ORYSJ	
18	RNA recognition motif	*Hordeum vulgare*	5,52E-05	0,0691901	0,0374703	0,541555801	decreased	PMF	73	30	5.82	41270	5.39	39976	gi|326487243	
19	Hypothetical protein OsI_14567	*Hordeum vulgare*	0,00195788	0,0835339	0,0550779	0,659347882	decreased	PMF	78	44	5.56	35629	5.66	40934	gi|218194204	
20	Ricin-type beta-trefoil (carbohydrate-binding domain)	*Hordeum vulgare*	0,00126349	0,0989736	0,240784	2,432810366	INCREASED	PMF	241	69	6.27	39056	6.95	40873	gi|326497973	
21	Unidentified	*Hordeum vulgare*	0,00863337	0,290597	1,26593	4,356307877	INCREASED						6.87	40632		A16
22	Chaperone protein	*Hordeum vulgare*	0,0196692	0,0588193	0,13181	2,240931123	INCREASED	PMF	113	56	5.15	31518	5.17	41116	gi|326506152	
23	Unidentified		3,99E-04	0,108509	0,0725019	0,668164853	decreased						6.13	42919		
24	Horseradish peroxidase/related secretory plant peroxidase	*Hordeum vulgare*	0,00152739	0,295669	0,14581	0,493152816	decreased	PMF	159	43	6.33	39594	6.78	44208	gi|326499758	
25	Pyruvate dehydrogenase E1 component subunit alpha-2, mitochondrial	*Arabidopsis thaliana*	7,01E-05	0,140073	0,568615	4,059419017	INCREASED	PMF	89	48	8.07	43666	6.08	44077	ODPA2_ARATH	
26	Fructokinase	*Hordeum vulgare*	0,00177889	0,788545	0,444275	0,563411093	decreased	PMF	163	62	5.06	35907	5.1	44339	gi|326513418	A1
27	Unidentified		0,00150196	0,118093	0,0573461	0,485601179	decreased						6.14	44077		
28	Superoxide dismutase [Mn] 3.3, mitochondrial	*Oryza sativa*	1,49E-04	0,57194	0,27362	0,478406826	decreased	MS/MS	63	8	5.97	25432	5.56	45000	SODM3_MAIZE	A7
29	Fructokinase	*Hordeum vulgare*	0,004465	0,247406	0,151199	0,611137159	decreased	PMF	152	58	5.67	34933	5.77	44933	gi|326489677	A2
30	Ricin-type beta-trefoil (carbohydrate-binding domain)	*Hordeum vulgare*	0,0120305	0,096452	0,0390415	0,404776469	decreased	PMF	113	38	5.71	35765	6.18	45591	gi|326517467	
31	Unidentified		0,00835077	0,0128511	0,0415294	3,231583289	INCREASED						4.55	50063		
32	Late-embryogenesis abundant protein	*Hordeum vulgare*	0,0160888	0,0629528	0,114668	1,821491657	INCREASED	PMF	102	44	4.96	36225	4.9	49846	gi|326528557	
33	Unidentified		6,14E-04	0,038385	0,0186672	0,486314967	decreased						4.76	51165		
34	Unidentified		0,0102831	0,0960149	0,205775	2,143156948	INCREASED						6.25	50832		
35	Unidentified		7,17E-04	0,0211916	0,0614182	2,898233262	INCREASED						4.75	61964		A15
36	Unidentified		1,10E-04	0,0711802	0,0492973	0,692570406	decreased						4.98	61964		
37	ATP synthase subunit beta, mitochondrial	*Hordeum vulgare*	0,00988942	0,12284	0,0796922	0,648747965	decreased	MS/MS	48	66	7.8	6430	5.29	69805	ATP5B_HORVU	
38	26S protease regulatory subunit 4 homolog	*Hordeum vulgare*	0,00299091	0,181946	0,0781122	0,429315291	decreased	PMF	112	38	5.91	49727	6.26	74735	PRS4_ORYSJ	
39	Betaine aldehyde dehydrogenase	*Hordeum vulgare*	0,016482	0,0331366	0,0970926	2,930071281	INCREASED	PMF	132	37	5.47	55276	5.84	80600	BADH_HORVU	A9
40	Unidentified		6,55972-04	0,0430649	0,261025	6,061200653	INCREASED						6.47	87137		
41	2,3-bisphosphoglycerate-independent phosphoglycerate mutase	*Mesembryanthemum crystallinum*	1,38E-06	0,0229434	0,202896	8,843327493	INCREASED	MS/MS	61	24	5.39	61316	6.25	88852	PNGI_MESCR	A5
42	NADP-dependent malic enzyme	*Oryza sativa*	1,54E-04	0,0280898	0,1073	3,819891918	INCREASED	MS/MS	39	1	6.7	70278	6.32	89723	MAOC_ORYSJ	A3
44	Heat shock 70 kDa protein	*Hordeum vulgare*	1,59E-04	0,48855	0,181222	0,370938491	decreased	PMF	136	45	5.14	71371	5.13	92611	gi|326519769	A11
45	Unidentified		9,25E-04	0,0785808	0,0370077	0,470950919	decreased						6.35	97474		
47	Unidentified		8,38E-04		0,095493		**INCREASED**						5.47	13580		
48	Unidentified		4,75E-04		0,120961		**INCREASED**						5.88	14059		
49	Unidentified		2,31E-04		0,0610428		**INCREASED**						4.68	14681		
50	Unidentified		3,51E-05		0,0763214		**INCREASED**						5.44	15837		
52	Universal stress protein 23267	*Hordeum vulgare*	0,00563157		0,0264643		**INCREASED**	PMF	61	52	6.30	17549	6.72	19079	gi|283970966	
54	Unidentified		1,19E-04		0,0817914		**INCREASED**						5.86	20715		A17
57	Dienelactone hydrolase	*Hordeum vulgare*	2,21E-04		0,0771355		**INCREASED**	PMF	70	34	5.06	25545	4.95	30855	gi|326491903	
58	Unidentified		1,40E-05		0,0421306		**INCREASED**						6.61	32352		
66	Unidentified		9,81E-06		0,0442386		**INCREASED**						6.40	37623		
67	GDSL esterase/lipase At4g10955	*Arabidopsis thaliana*	2,43E-04		0,0371127		**INCREASED**	MS/MS	35	2	7.88	39563	6.27	38524	GDL62_ARATH	
69	Unidentified		0,00128447		0,0267693		**INCREASED**						6.52	40154		
70	Unidentified		0,00465415		0,228762		**INCREASED**						6.77	41177		
71	Seed maturation protein	*Hordeum vulgare*	0,00193751		0,0988531		**INCREASED**	PMF	60	29	4.27	26900	4.03	40873	gi|326512936	
73	Hypotetical conserved protein Os10g0395500	*Oryza sativa*	2,83E-04		0,105319		**INCREASED**	PMF	81	53	7.85	9299	6.49	46090	gi|297610438	
74	Ricin-type beta-trefoil lectin domain-like	*Hordeum vulgare*	1,18E-07		0,0298951		**INCREASED**	PMF	163	46	5.71	35765	6.03	46392	gi|326517467	
75	Unidentified		4,28E-04		0,102036		**INCREASED**						6.78	52978		
76	Pyruvate dehydrogenase E1 component subunit alpha-2, mitochondrial	*Arabidopsis thaliana*	7,72E-04		0,0960126		**INCREASED**	PMF	89	48	8.07	43666	5.75	52748	ODPA2_ARATH	
77	Horseradish peroxidase/related secretory plant peroxidase	*Hordeum vulgare*	5,87E-04		0,0513642		**INCREASED**	PMF	86	29	6.31	36229	6.58	53093	gi|326513264	
80	Unidentified		2,73E-08		0,0889129		**INCREASED**						4.87	60104		
81	Unidentified		1,04E-05		0,0555486		**INCREASED**						6.14	65144		
82	Ankyrin repeats containing protein	*Hordeum vulgare*	5,36E-07		0,0531833		**INCREASED**	PMF	80	35	4.58	39949	4.48	65857	gi|326520599	
84	Glucose- 6-phosphate dehydrogenase, cytosolic	*Hordeum vulgare*	8,10E-04		0,0333952		**INCREASED**	PMF	76	27	6.27	58066	6.46	81191	gi|259166850	
85	Pyruvate decarboxylase isozyme 1	*Zea mays*	1,91E-05		0,0424037		**INCREASED**	MS/MS	48	1	6.47	66355	6.16	83805	PDC1_MAIZE	A4
86	Heat shock 70 kDa protein	*Zea mays*	7,99E-05		0,335572		**INCREASED**	MS/MS	224	5	5.22	70871	5.16	94204	HSP70_MAIZE	
89	Unidentified		0,032981	0,0281771			**decreased**						5.65	15265		
91	Unidentified		0,0158767	0,0405581			**decreased**						4.97	30312		
92	Unidentified		1,51E-06	0,0401687			**decreased**						5.40	34172		

93	Unidentified		0,007795	0,0474488			**decreased**						6.20	42101		
94	Unidentified		2,37E-05	0,125123			**decreased**						4.82	47619		
95	Unidentified		0,0136673	0,0452498			**decreased**						4.85	69635		
96	Lactoylglutathione lyase	*Arabidopsis thaliana*	0,0115421	0,498878	0,687915	1,378924306	INCREASED	MS/MS	55	3	7.7	39427	5.31	40572	LGUC_ARATH	A6
97	Cold shock protein	*Hordeum vulgare*	0,0130303	0,125247	0,168735	1,347217897	INCREASED	PMF	124	68	5.94	21903	6.40	25228	gi|326523669	
98	Ascorbate peroxidase 2, cytosolic	*Oryza sativa*	0,0124782	0,647327	0,476997	0,736871782	decreased	PMF	117	52	5.10	27964	6.20	32457	gi|15808779	A8
99	Betaine aldehyde dehydrogenase	*Hordeum vulgare*	0,0086807	0,112754	0,174987	1,551936073	INCREASED	PMF	123	33	5.82	55098	5.89	74589	BADH_HORVU	
