# Supplementary material for: Analysis of Drought-Induced Proteomic and Metabolomic Changes in Barley (Hordeum vulgare L.) Leaves and Roots Unravels Some Aspects of Biochemical Mechanisms Involved in Drought Tolerance
Source: Front Plant Sci. 2016 Jul 26;7:1108. doi: 10.3389/fpls.2016.01108 (PMC4962459; doi:10.3389/fpls.2016.01108)
Supplement: Supplementary file 8 [file Table_3.PDF]

Table S3. List of drought-responsive proteins in Cam/B1/CI root extracts.

Spot no.	Protein name	Organism	ANOVA	CAM control	CAM stress	Stress/Control	Accumulation level	Method of identification	Score	% coverage	pI (theoretical)	Mw (theoretical)	pI (experimental)	Mw (experimental)	Accession number	Position on the gel
1	Unidentified		0,0393685	0,173431	0,327803	1,890106152	INCREASED						4.39	13757		
2	Unidentified		0,00199635	0,0274482	0,070168	2,556378925	INCREASED						4.64	14491		
3	Heat shock 17.6 kDa protein 3	*Arabidopsis thaliana*	0,024053	0,0406494	0,0949252	2,33521774	INCREASED	MS/MS	148	15	5.36	17593	5.34	16502	HS17C_ARATH	A12
4	Unidentified		0,0622283	0,0376606	0,108685	2,885907288	INCREASED						5.44	16754		
5	Cold-regulated protein	*Hordeum vulgare*	0,00622271	0,124259	0,242579	1,952204669	INCREASED	PMF	65	46	4.93	17659	4.79	16682	gi|10799810	A10
6	Peroxiredoxin family	*Hordeum vulgare*	0,00534136	0,0576222	0,081779	1,419227312	INCREASED	PMF	111	54	9.04	24204	4.86	17121	gi|326497111	
7	Unidentified		0,00154504	0,102289	0,055486	0,542443469	decreased						6.17	17383		
8	Unidentified		0,0100144	0,00969735	0,0713367	7,356308682	INCREASED						4.13	19038		
9	Translation initiation factor 5A	*Secale cereale*	0,00157744	0,193678	0,125562	0,648302853	decreased	PMF	72	66	5.03	12618	5.74	19162	gi|82754830	
10	Unidentified		8,25E-04	0,0712444	0,0526506	0,739013873	decreased						5.57	24054		
11	Unidentified		0,0386089	0,0399557	0,0784204	1,962683672	INCREASED						4.49	25669		
12	Thioredoxin peroxidase	*Hordeum vulgare*	0,00594081	0,0617811	0,0822967	1,332069193	INCREASED	PMF	90	41	6.33	28441	4.76	25724	gi|326496957	
13	Unidentified		3,32E-04	0,108863	0,07184	0,659911999	decreased						4.93	26805		
14	Adenine phosphoribosyltransferase	*Hordeum vulgare*	0,00660761	0,104029	0,0769214	0,739422661	decreased	PMF	131	55	5.15	19777	4.97	28052	gi|9711921	
15	Unidentified		0,0106014	0,0551437	0,0330985	0,600222691	decreased						4.49	27810		
16	Oxalate oxidase 1	*Hordeum vulgare*	0,0155097	0,0732704	0,149929	2,046242412	INCREASED	MS/MS	175	12	5.52	21304	6.16	27511	OXO1_HORVU	
17	Unidentified		0,0165122	0,0941206	0,0629475	0,668796204	decreased						5.20	30044		
18	Unidentified		0,024952	0,0213452	0,0602811	2,824105654	INCREASED						5.16	30357		
19	Ascorbate peroxidase 2, cytosolic	*Hordeum vulgare*	0,0017129	0,627902	0,414427	0,660018602	decreased	PMF	117	52	5.10	27964	5.03	31501	gi|15808779	A8
20	S-adenosylmethionine-dependent methyltransferase	*Hordeum vulgare*	0,0198684	0,283239	0,208375	0,735686117	decreased	PMF	80	52	4.92	27313	4.92	31642	gi|326494234	
21	Unidentified		0,0314016	0,063409	0,0809916	1,277288713	INCREASED						5.58	33029		
22	Unidentified		0,0256069	0,0586403	0,0183897	0,313601738	decreased						6.30	34375		
23	Isopentenyl diphosphate isomerase	*Hordeum vulgare*	0,0123418	0,0729693	0,0546818	0,749380904	decreased	PMF	135	59	4.99	27252	4.92	35513	gi|326508098	
24	P-loop containing nucleoside triphosphate hydrolase	*Hordeum vulgare*	0,0396562	0,0428185	0,0921934	2,153120731	INCREASED	PMF	114	40	7.08	38699	6.06	35356	gi|326522022	A14
25	Phytepsin	*Hordeum vulgare*	0,0131229	0,107051	0,0762713	0,712476296	decreased	PMF	112	35	5.28	51932	4.97	36797	ASPR_HORVU	
26	Unidentified		0,0427176	0,046143	0,0982286	2,128786598	INCREASED						5.23	38581		
27	Caffeoyl-CoA O-methyltransferase 1	*Zea mays*	3,15E-04	0,110915	0,070012	0,631222107	decreased	MS/MS	49	3	5.14	29016	4.98	39638	CAMT1_MAIZE	
28	Soluble NSF attachment protein family	*Hordeum vulgare*	9,94E-04	0,0681129	0,0550952	0,80888055	decreased		74		4.92	32773	4.87	38983	gi|326502724	
29	Lactoylglutathione lyase	*Arabidopsis thaliana*	0,0470642	0,486842	0,566755	1,164145657	INCREASED	MS/MS	55	3	7.7	39427	5.27	39858	LGUC_ARATH	A6
30	Unidentified		0,005353	0,416301	0,857097	2,058839638	INCREASED						6.87	39976		A16
31	Esterase/lipase family	*Hordeum vulgare*	0,00794025	0,152824	0,101144	0,66183322	decreased	PMF	105	41	5.46	36937	5.49	40213	gi|326502616	
32	Aldo-keto reductase family	*Hordeum vulgare*	0,0131704	0,12749	0,0831726	0,652385285	decreased	PMF	80	21	6.19	37380	6.17	40392	gi|326514718	
33	Isoflavone reductase-like	*Hordeum vulgare*	0,0379977	0,0850892	0,0664631	0,781099129	decreased	PMF	103	40	6.16	36066	6.24	40392	gi|197709162	
34	Fructokinase	*Hordeum vulgare*	0,00367136	0,612142	0,276324	0,451405066	decreased	PMF	137	48	5.06	35907	5.07	43430	gi|326513418	A1
35	Ricin-type beta-trefoil (carbohydrate-binding domain)	*Hordeum vulgare*	9,95E-06	0,187497	0,335849	1,791223326	INCREASED	PMF	137	38	5.71	35765	6.16	42919	gi|326517467	
36	Unidentified		0,0155646	0,0424695	0,0597017	1,405754718	INCREASED						6.40	43110		
37	Ricin-type beta-trefoil (carbohydrate-binding domain)	*Hordeum vulgare*	0,0467904	0,0658806	0,131797	2,000543407	INCREASED	PMF	131	38	5.71	35765	6.07	43110	gi|326517467	
38	Unidentified		0,00764369	0,094366	0,0628212	0,665718585	decreased						5.11	45295		
39	Unidentified		7,27E-05	0,318308	0,150291	0,472155899	decreased						5.68	45691		
40	Malate dehydrogenase, cytoplasmic	*Hordeum vulgare*	0,00746443	1,06001	0,676098	0,637822285	decreased	PMF	99	45	5.62	35920	5.82	45295	F2D4W6_HORVD	
41	Pyruvate dehydrogenase E1 component beta subunit, mitochondrial	*Arabidopsis thaliana*	0,0137369	0,893489	0,629186	0,704189979	decreased	MS/MS	45	4	5.67	39436	4.92	46392	ODPB_ARATH	
42	Unidentified		0,0362391	0,0127069	0,0377521	2,970992138	INCREASED						4.51	47619		
43	Unidentified		3,86E-05	0,0614679	0,108796	1,769964486	INCREASED						5.23	51054		
44	S-adenosylmethionine synthase 2	*Hordeum vulgare*	0,00444153	0,651934	0,330615	0,507129556	decreased	MS/MS	436	14	5.58	43257	5.91	55940	METK2_HORVU	
45	S-adenosylmethionine synthase	*Hordeum vulgare*	0,0128765	1,19833	0,638789	0,533066017	decreased	PMF	126	45	5.51	43138	5.76	55455	METK3_HORVU	
46	Unidentified		0,0070659	0,0228187	0,0751518	3,293430388	INCREASED						5.27	58811		
47	Unidentified		0,0105699	0,0318338	0,0592284	1,860550735	INCREASED						4.70	59196		A15
48	Unidentified		0,00212667	0,00582445	0,0419998	7,210946956	INCREASED						6.01	60894		
49	Unidentified		0,0117096	0,0587843	0,1083	1,842328649	INCREASED						6.78	62642		
50	Unidentified		0,00148379	0,242032	0,097655	0,403479705	decreased						5.04	63880		
51	UTP-glucose-1-phosphate uridylyltransferase	*Hordeum vulgare*	0,0345014	0,546478	0,401324	0,73438272	decreased	PMF	151	45	5.20	51783	5.17	65286	UGPA_HORVU	
52	26S protease regulatory subunit 6A homolog	*Oryza sativa*	8,23E-04	0,0974805	0,0623711	0,639831556	decreased	PMF	88	48	5.51	21851	4.74	65428	PRS6A_ORYSJ	
53	26S protease regulatory subunit 6A homolog	*Brassica campestris*	4,21E-04	0,0875714	0,0317725	0,362818226	decreased	PMF	176	38	4.97	48557	4.82	65286	PRS6A_BRACM	
54	Unidentified		0,00192444	0,467724	2,99426	6,401766854	INCREASED						6.68	66465		
55	Unidentified		0,00294868	0,250147	0,954659	3,816391962	INCREASED						6.55	64720		
56	Unidentified		0,00242169	0,154069	0,450194	2,922028442	INCREASED						6.42	66000		

57	Unidentified		0,00449302	0,0766749	0,0402314	0,524701043	decreased						4.87	69128		
58	Unidentified		0,00757271	0,0846614	0,0432772	0,511179829	decreased						4.75	69128		
59	Unidentified		0,00673717	0,181592	0,585317	3,223253227	INCREASED						6.47	66322		
60	Unidentified		9,46E-04	0,11217	0,0465433	0,414935366	decreased						6.27	71179		
61	Unidentified		0,00807563	0,159421	0,221894	1,391874345	INCREASED						6.12	74010		
62	Delta-1-pyrroline-5-carboxylate dehydrogenase 12A1, mitochondrial	*Arabidopsis thaliana*	0,00110004	0,0607098	0,0822293	1,354465012	INCREASED	MS/MS	137	6	6.26	62190	6.42	75283	AL121_ARATH	
63	Betaine aldehyde dehydrogenase	*Hordeum vulgare*	0,0142013	0,0866563	0,132864	1,533229552	INCREASED	PMF	123	33	5.82	55098	6.20	74735	BADH_HORVU	A9
64	Unidentified		0,0132435	0,0672822	0,0897678	1,334198347	INCREASED						6.31	77140		
65	Pyruvate decarboxylase isozyme 1	*Zea mays*	8,98E-04	0,00331828	0,0257899	7,772068662	INCREASED	MS/MS	48	1	6.47	66355	6.16	80208	PDC1_MAIZE	A4
66	2,3-bisphosphoglycerate-independent phosphoglycerate mutase	*embryanthemum crystalli*	0,00279393	0,0310716	0,0977233	3,145100349	INCREASED	MS/MS	61	24	5.39	61316	6.25	85454	PNGI_MESCR	A5
67	NADP-dependent malic enzyme	*Hordeum vulgare*	0,0118067	0,046995	0,115986	2,468049793	INCREASED	PMF	122	26	6.46	68655	5.99	85246	gi|326530055	
68	Phosphoglucomutase 1	*Hordeum vulgare*	0,00689515	0,170774	0,135846	0,795472379	decreased	PMF	218	49	5.28	62954	5.49	87137	gi|326504468	
69	Vacuolar proton-ATPase	*Hordeum vulgare*	0,0053229	0,122671	0,0937407	0,764163494	decreased	PMF	241	52	5.23	68752	5.30	87349	gi|11527563	
70	NADP-dependent malic enzyme	*Oryza sativa*	0,0208607	0,0244886	0,0575137	2,348590773	INCREASED	MS/MS	39	1	6.7	70278	6.32	86292	MAOC_ORYSJ	A3
71	Heat shock 70 kDa protein	*Vigna radiata*	8,97E-04	0,389569	0,197771	0,507666164	decreased	PMF	74	26	5.14	71583	5.09	88420	gi|45331283	A11
72	Unidentified		0,0133292	0,0372536	0,178061	4,779699143	INCREASED						6.15	93518		
73	Unidentified		5,54E-04	0,0445101	0,107921	2,4246407	INCREASED						5.95	90161		
74	Unidentified		0,003221	0,0402743	0,0694491	1,72440241	INCREASED						5.63	92837		
75	Phenylalanine ammonia-lyase	*Oryza sativa*	2,55E-06	0,264662	0,0943125	0,356350742	decreased	PMF	78	30	5.73	54553	6.25	93518	PAL1_ORYSJ	
76	Heat shock 70 kDa protein	*Spinacia oleracea*	0,00896108	0,111939	0,154864	1,383467782	INCREASED	MS/MS	80	2	4.87	64918	4.60	94434	HSP7S_SPIOL	
77	Phenylalanine ammonia-lyase	*Hordeum vulgare*	6,23E-04	0,0738	0,0363479	0,49251897	decreased	PMF	109	32	5.89	76199	6.36	9204	gi|326526355	
78	Unidentified		0,00206004	0,0258385	0,08389	3,246705498	INCREASED						5.36	95592		
79	Transketolase	*Zea mays*	0,0026182	0,0848742	0,131112	1,544780393	INCREASED	MS/MS	47	1	5.47	73347	5.49	95359	TKTC_MAIZE	
80	Transketolase	*Zea mays*	0,0161628	0,0451897	0,0801987	1,774711937	INCREASED	MS/MS	49	1	5.47	73347	5.42	95825	TKTC_MAIZE	
82	Chaperone protein ClpC1	*Brachypodium distachyo*	0,00328177	0,0429924	0,062799	1,460700031	INCREASED	PMF	113	17	5.95	97689	5.59	103345	gi|357149201	
83	Lipoxygenase	*Hordeum vulgare*	0,00395998	0,121565	0,16964	1,395467445	INCREASED	PMF	91	17	5.73	96447	6.17	104104	LOX1_HORVU	
84	Unidentified		1,29E-04	0,171442	0,756973	4,415329966	INCREASED						6.61	65002		
85	Unidentified		0,011692	0,513667	0,319268	0,621546644	decreased						5.03	87990		
86	Molecular chaperone DnaK	*Hordeum vulgare*	0,00183904	0,361328	0,209554	0,579955055	decreased	PMF	124	32	5.54	73034	6.03	99393	gi|326514246	
87	Heat shock 16.9 kDa protein 1	*Hordeum vulgare*	0,0330392	0,164344	0,569734	3,466716156	INCREASED	PMF	89	49	5.83	16917	5.77	15298	gi|326490111	A13
88	Unidentified		0,00530183	0,114979	0,0872071	0,75846111	decreased						5.16	36960		
89	Unidentified		0,0295595	0,095687	0,0670872	0,701110914	decreased						5.31	27273		
90	Glutathione S-transferase	*Hordeum vulgare*	0,00581422	0,0713723	0,0319047	0,447017961	decreased	PMF	80	29	5.41	25132	5.68	32018	gi|326501538	
91	Phenylalanine ammonia-lyase	*Oryza sativa*	9,64E-04	0,0756543	0,0332087	0,438953239	decreased	MS/MS	109	3	6.07	76021	6.13	94665	PAL1_ORYSJ	
92	Heat shock 70 kDa protein	*Hordeum vulgare*	0,0194	0,073159	0,0415854	0,568424937	decreased	PMF	113	34	5.14	71620	4.97	87900	gi|224098390	
94	Unidentified		0,00265154		0,0535949		**INCREASED**						5.83	20537		A17
95	Unidentified		8,05E-06		0,0797911		**INCREASED**						5.72	28358		
96	2-dehydro-3-deoxyphosphoheptonate aldolase	*Morinda citrifolia*	1,24E-06		0,0289863		**INCREASED**	PMF	73	35	7.28	59529	6.67	35251	gi|2546988	
97	Unidentified		0,00383016		0,0203687		**INCREASED**						6.41	36906		
99	Unidentified		0,00228152		0,047719		**INCREASED**						6.74	74917		
100	NAD+-dependent alpha-aminoadipic semialdehyde dehydrogenase	*Hordeum vulgare*	2,69E-06		0,0244509		**INCREASED**	PMF	96	32	5.77	55081	6.09	77517	gi|326510533	
101	Unidentified		0,00628129		0,0486647		**INCREASED**						6.32	91267		
102	Unidentified		3,62E-04	0,0489992		0	**decreased**						5.69	15397		
103	Unidentified		0,00234215	0,0218523		0	**decreased**						5.65	28978		
104	Flavonoid O-methyltransferase	*Hordeum vulgare*	1,14E-04	0,0297825		0	**decreased**	PMF	116	34	5.64	38982	5.90	44933	gi|148337324	
105	Unidentified		0,00153462	0,0621557	0,01296238	0,208546923	decreased						4.90	79623		
106	Fructokinase	*Hordeum vulgare*	1,61E-03		0,122063	0	**INCREASED**	PMF	152	58	5.67	34933	5.57	81588	gi|326489677	A2
107	Superoxide dismutase [Mn] 3.3, mitochondrial	*Oryza sativa*	0,025563	0,518249	0,370556	0,715015369	decreased	MS/MS	57	8	5.97	25432	5.56	45689	SODM3_MAIZE	A7
