# Supplementary material for: Analysis of Drought-Induced Proteomic and Metabolomic Changes in Barley (Hordeum vulgare L.) Leaves and Roots Unravels Some Aspects of Biochemical Mechanisms Involved in Drought Tolerance
Source: Front Plant Sci. 2016 Jul 26;7:1108. doi: 10.3389/fpls.2016.01108 (PMC4962459; doi:10.3389/fpls.2016.01108)
Supplement: Supplementary file 9 [file Table_4.PDF]

Table S4. List of drought-responsive proteins in Maresi leaf extracts.

| Spot no. | Protein name | *Organism* | ANOVA | MARESI control | Maresi stress | Stress/Control | Accumulation level | Method of identification | Score | % coverage | pI (theoretical) | Mw (theoretical) | Mw (experimental) | pI (experimental) | Accession number | Position on the gel |
|---|---|---|---|---|---|---|---|---|---|---|---|---|---|---|---|---|
| 1 | 2-Cys peroxiredoxin BAS1, chloroplastic | *Hordeum vulgare* | 0,00824781 | 0,0156826 | 0,0526933 | 3,359984951 | INCREASED | PMF | 82 | 40 | 5.4 | 23398 | 11494 | 6.44 | BAS1_HORVU | A8 |
| 2 | Unidentified | | 0,00924878 | 0,0151548 | 0,0556934 | 3,674967667 | INCREASED | | | | | | 11794 | 5.34 | | |
| 3 | Delta-1-pyrroline-5-carboxylate synthetase | *Vigna aconitifolia* | 0,017887 | 0,0518764 | 0,0252025 | 0,485818214 | decreased | MS/MS | 33 | 1 | 6.6 | 73531 | 32575 | 5.6 | P5CS_VIGAC | |
| 4 | Cytochrome B6-F complex Fe-S subunit | *Hordeum vulgare* | 0,00246835 | 0,0452852 | 0,117158 | 2,587114554 | INCREASED | PMF | 74 | 22 | 8.47 | 24124 | 14400 | 5.86 | gi\|326518434 | |
| 5 | Unidentified | | 0,0124549 | 0,0161897 | 0,0423942 | 2,618590832 | INCREASED | | | | | | 19744 | 6.16 | | |
| 6 | Probable mannitol dehydrogenase | *Maedicago sativa* | 0,0412959 | 0,0178114 | 0,0434522 | 2,439572409 | INCREASED | MS/MS | 30 | 2 | 7.8 | 39458 | 20351 | 4.67 | MTDH_MEDSA | |
| 9 | Ribosomal protein L10 family | *Hordeum vulgare* | 0,0382794 | 0,0690606 | 0,0519842 | 0,752733107 | decreased | PMF | 121 | 43 | 8.31 | 24197 | 24566 | 5.99 | gi\|326507838 | |
| 10 | Glutathione S-transferase | *Hordeum vulgare* | 3,10E-04 | 0,0390441 | 0,0769928 | 1,971944545 | INCREASED | PMF | 71 | 37 | 5.67 | 23557 | 27108 | 6.25 | gi\|326490728 | |
| 11 | 2-Cys peroxiredoxin BAS1, chloroplastic | *Hordeum vulgare* | 0,00135921 | 0,0259449 | 0,0712672 | 2,7468674 | INCREASED | MS/MS | 101 | 15 | 5.4 | 23398 | 26718 | 4.79 | BAS1_HORVU | |
| 12 | Unidentified | | 0,0057339 | 0,0136764 | | 0 | **decreased** | | | | | | 28559 | 6.32 | | |
| 13 | Photosystem II oxygen-evolving complex protein 2 precursor | *Triticum aestivum* | 0,00703619 | 0,0947474 | 0,174423 | 1,840926506 | INCREASED | PMF | 110 | 41 | 8.84 | 27424 | 30038 | 5.51 | PSBP_WHEAT | |
| 14 | Unidentified | | 0,00777869 | 0,157115 | 0,105868 | 0,673824905 | decreased | | | | | | 30498 | 5.24 | | |
| 15 | Signal recognition particle protein 1 | *Arabidopsis thaliana* | 0,0190491 | 0,0667629 | 0,0410989 | 0,615594889 | decreased | MS/MS | 29 | 1 | 9.7 | 53257 | 31123 | 5.09 | SR5541_ARATH | |
| 16 | Ascorbate peroxidase | *Hordeum vulgare* | 1,65E-05 | 0,0185057 | 0,0448493 | 2,423539774 | INCREASED | PMF | 80 | 34 | 5.85 | 27532 | 31801 | 6.35 | gi\|3688398 | |
| 18 | Glutathione S-transferase | *Hordeum vulgare* | 0,00259258 | 0,0110753 | 0,0391961 | 3,539055375 | INCREASED | PMF | 112 | 34 | 8.91 | 33484 | 33709 | 5.56 | gi\|326507956 | A7 |
| 19 | Ubiquitin carboxyl-terminal hydrolase 19 | *Arabidopsis thaliana* | 0,00112161 | 0,0773346 | 0,0381097 | 0,492789773 | decreased | MS/MS | 28 | 1 | 4.8 | 77097 | 34750 | 5.15 | UBP19_ARATH | A9 |
| 20 | RNA recognition motif | *Hordeum vulgare* | 0,00440885 | 0,0242254 | 0,0786382 | 3,246105327 | INCREASED | PMF | 112 | 40 | 4.57 | 31872 | 35687 | 4.33 | gi\|326493824 | |
| 21 | Oxygen-evolving enhancer protein 1, chloroplastic | *Solanum tuberosum* | 4,15E-04 | 0,446458 | 0,931164 | 2,085669873 | INCREASED | MS/MS | 330 | 13 | 5.84 | 35595 | 37164 | 5.06 | PSBO_SOLTU | |
| 22 | Unidentified | | 2,05E-04 | 0,0239574 | | 0 | **decreased** | | | | | | 38263 | 5.88 | | |
| 23 | Oxygen-evolving enhancer protein 1, chloroplastic | *Solanum tuberosum* | 0,0019328 | 0,0671474 | 0,0328832 | 0,489716653 | decreased | MS/MS | 164 | 8 | 5.84 | 35595 | 39195 | 5.27 | PSBO_SOLTU | |
| 24 | Ferredoxin-NADP+ oxidoreductase | *Hordeum vulgare* | 2,29E-05 | 0,171314 | 0,0857088 | 0,500302369 | decreased | PMF | 90 | 32 | 8.29 | 39971 | 40457 | 6.37 | gi\|326492141 | A10 |
| 25 | Ferredoxin-NADP+ oxidoreductase | *Triticum aestivum* | 0,00877661 | 0,221392 | 0,159515 | 0,720509323 | decreased | PMF | 104 | 33 | 6.92 | 40491 | 39695 | 5.83 | gi\|20302473 | |
| 26 | Unidentified | | 9,99E-04 | 0,0540285 | 0,16996 | 3,145747152 | INCREASED | | | | | | 41128 | 5.16 | | A14 |
| 28 | Cysteine synthase, mitochondrial | *Hordeum vulgare* | 0,00317391 | 0,138881 | 0,078134 | 0,562596755 | decreased | MS/MS | 46 | 3 | 5.3 | 34207 | 41285 | 5.28 | CYSK_HORVU | |
| 29 | Unidentified | | 0,0112754 | 0,0413168 | 0,0292089 | 0,706949715 | decreased | | | | | | 42077 | 6.16 | | |
| 30 | Cysteine synthase | *Triticum aestivum* | 1,85E-05 | 0,0604608 | | 0 | **decreased** | PMF | 85 | 40 | 5.48 | 34207 | 41600 | 5.75 | CYSK_WHEAT | |
| 31 | Ricin-type beta-trefoil (carbohydrate-binding domain) | *Hordeum vulgare* | 2,58E-04 | 0,0453268 | | 0 | **decreased** | PMF | 92 | 39 | 5.71 | 35765 | 42885 | 6.07 | gi\|326517467 | |
| 33 | Unidentified | | 2,01E-04 | 0,0334643 | 0,0768129 | 2,295368497 | INCREASED | | | | | | 44041 | 6.06 | | |
| 34 | Unidentified | | 0,0180996 | 0,317756 | 0,586848 | 1,846851043 | INCREASED | | | | | | 45318 | 6.18 | | |
| 35 | Nucleoside-diphosphate-sugar epimerase | *Hordeum vulgare* | 0,0108349 | 0,362165 | 0,256424 | 0,70803087 | decreased | PMF | 179 | 42 | 7.06 | 41582 | 45212 | 6.02 | gi\|326523629 | |
| 36 | Unidentified | | 0,0188379 | 0,052239 | 0,022937 | 0,439078083 | decreased | | | | | | 46834 | 5.94 | | |
| 37 | Dynamin-related protein 1B | *Arabidopsis thaliana* | 0,02163 | 0,0732544 | 0,0314158 | 0,428858881 | decreased | PMF | 77 | 39 | 7.64 | 68384 | 46724 | 5.8 | T47968 | |
| 38 | Ribulose bisphosphate carboxylase/oxygenase activase B, chloroplastic | *Triticum aestivum* | 3,22E-04 | 0,0224043 | 0,105816 | 4,72302192 | INCREASED | PMF | 145 | 41 | 6.92 | 48012 | 47387 | 5.53 | gi\|7960277 | A3 |
| 39 | Fructose-1,6-bisphosphate aldolase | *Hordeum vulgare* | 0,00516084 | 0,309251 | 0,236565 | 0,764961148 | decreased | PMF | 198 | 53 | 6.06 | 38102 | 49901 | 6.37 | gi\|326493652 | |
| 40 | Unidentified | | 0,00517168 | 0,0990728 | 0,0588399 | 0,593905694 | decreased | | | | | | 54306 | 6.31 | | |
| 41 | Glyceraldehyde-3-phosphate dehydrogenase A, chloroplastic | *Arabidopsis thaliana* | 0,00481321 | 0,671513 | 0,38146 | 0,568060484 | decreased | MS/MS | 121 | 3 | 9.8 | 40507 | 52425 | 5.83 | G3PB_ARATH | |
| 42 | Glutamine synthetase leaf isozyme, chloroplastic | *Hordeum vulgare* | 1,54E-04 | 1,34268 | 0,839211 | 0,625026812 | decreased | PMF | 119 | 40 | 5.75 | 46902 | 53295 | 5.11 | gi\|755762 | A11 |
| 43 | ATP-sulfurylase | *Hordeum vulgare* | 0,00290441 | 0,0673919 | 0,0431533 | 0,640333631 | decreased | PMF | 102 | 31 | 5.95 | 39484 | 58684 | 6.33 | gi\|326530496 | |
| 44 | Unidentified | | 0,042668 | 0,0477802 | 0,0234999 | 0,491833437 | decreased | | | | | | 56519 | 5.88 | | |
| 45 | Elongation factor Tu, chloroplastic-like | *Brachypodium distachyon* | 0,00381689 | 0,629716 | 0,342491 | 0,543881686 | decreased | PMF | 220 | 52 | 5.88 | 50638 | 56519 | 5.44 | gi\|357149925 | |
| 46 | Ribulose bisphosphate carboxylase/oxygenase activase A, chloroplastic | *Hordeum vulgare* | 0,0151096 | 0,363115 | 0,229185 | 0,631163681 | decreased | MS/MS | 441 | 14 | 8.04 | 51383 | 58822 | 5.23 | RCAA_HORVU | |
| 47 | S-adenosylmethionine synthase 3 | *Hordeum vulgare* | 2,92E-04 | 0,0292473 | | 0 | **decreased** | PMF | 122 | 42 | 5.51 | 43138 | 58409 | 5.70 | METK3_HORVU | |
| 48 | Ribulose bisphosphate carboxylase/oxygenase large subunit | *Hordeum vulgare* | 0,00171349 | 0,360107 | 0,135014 | 0,374927452 | decreased | MS/MS | 346 | 8 | 6.22 | 53672 | 64014 | 6.72 | RBL_HORVU | |
| 49 | Phosphogluconate dehydrogenase, cytosolic | *Zea mays* | 0,00171921 | 0,0094449 | | 0 | **decreased** | PMF | 93 | 25 | 5.61 | 52860 | 60790 | 5.77 | gi\|357110692 | |
| 50 | Enolase | *Hordeum vulgare* | 0,00545875 | 0,204331 | 0,123793 | 0,605845417 | decreased | PMF | 132 | 39 | 5.39 | 48601 | 69384 | 5.57 | gi\|326490934 | A4 |
| 51 | Ribulose bisphosphate carboxylase/oxygenase large subunit-binding protein su | *Secale cereale* | 0,016229 | 0,116266 | 0,0110193 | 0,094776633 | decreased | MS/MS | 108 | 3 | 4.88 | 53721 | 74045 | 5.05 | RUBB_SECCE | |
| 52 | Unidentified | | 1,52E-04 | 0,0181794 | | 0 | **decreased** | | | | | | 77196 | 6.25 | | |
| 53 | Ribulose bisphosphate carboxylase/oxygenase large subunit-binding protein su | *Secale cereale* | 5,77E-05 | 0,650508 | 0,245602 | 0,377554158 | decreased | MS/MS | 376 | 11 | 4.7 | 53721 | 76555 | 5.22 | RUBB_SECCE | A2 |
| 56 | Ferredoxin-nitrite reductase | *Hordeum vulgare* | 0,0338891 | 0,0987507 | 0,0542454 | 0,549316612 | decreased | PMF | 122 | 20 | 6.33 | 66660 | 78887 | 6.32 | gi\|326505210 | A12 |
| 57 | Ferredoxin-nitrite reductase | *Triticum aestivum* | 2,71E-05 | 0,0125165 | | 0 | **decreased** | PMF | 118 | 26 | 6.33 | 66660 | 78624 | 5.95 | gi\|326505210 | |
| 58 | Unidentified | | 7,92E-04 | 0,0151719 | | 0 | **decreased** | | | | | | 78493 | 5.90 | | |
| 59 | 26S proteasome non-ATPase regulatory subunit 7-like | *Brachypodium distachyon* | 7,36E-04 | 0,0198593 | | 0 | **decreased** | PMF | 77 | 36 | 6.23 | 34848 | 78624 | 5.70 | gi\|357166548 | |
| 60 | Unidentified | | 0,0170087 | 0,0847318 | 0,0548719 | 0,647595118 | decreased | | | | | | 81290 | 5.19 | | |
| 61 | Unidentified | | 5,91E-04 | 0,0175965 | 0,00193432 | 0,109926406 | decreased | | | | | | 82794 | 5.95 | | |
| 62 | Unidentified | | 0,00192781 | 0,0120464 | | 0 | **decreased** | | | | | | 84749 | 5.69 | | |
| 63 | Unidentified | | 0,00236225 | 0,023326 | 0,014623 | 0,626897025 | decreased | | | | | | 85174 | 5.65 | | |
| 64 | Heat shock 70 kDa protein, chloroplastic | *Hordeum vulgare* | 4,59E-04 | 0,144195 | 0,0875261 | 0,606998162 | decreased | PMF | 94 | 18 | 5.04 | 73955 | 87768 | 4.85 | gi\|326492960 | A6 |
| 65 | Thiamine pyrophosphate (TPP) family, transketolase subfamily | *Hordeum vulgare* | 0,00300857 | 0,488119 | 0,20419 | 0,418320123 | decreased | PMF | 163 | 37 | 5.45 | 74032 | 86750 | 5.62 | gi\|326533372 | |
| 66 | Thiamine pyrophosphate (TPP) family, transketolase subfamily | *Hordeum vulgare* | 0,0109381 | 0,349433 | 0,196495 | 0,562325253 | decreased | PMF | 124 | 30 | 5.45 | 74032 | 87185 | 5.54 | gi\|326533372 | |
| 67 | Methionine synthase | *Hordeum vulgare* | 0,00456423 | 0,0367712 | 0,0190693 | 0,518593356 | decreased | PMF | 185 | 29 | 5.68 | 84794 | 87914 | 5.94 | gi\|50897038 | |
| 69 | Chaperone protein ClpC1, chloroplastic | *Medicago truncatula* | 7,72E-04 | 0,133802 | 0,0438525 | 0,327741738 | decreased | PMF | 145 | 26 | 6.44 | 102889 | 93507 | 5.63 | gi\|357520587 | |
| 70 | Chaperone protein ClpC1, chloroplastic | *Oryza sativa* | 0,00844034 | 0,067875 | 0,0274875 | 0,404972376 | decreased | PMF | 233 | 30 | 6.14 | 101853 | 93663 | 5.58 | CLPC1_ORYSJ | |
| 71 | Clp protease ATP binding subunit | *Hordeum vulgare* | 0,00191313 | 0,0987698 | 0,0487688 | 0,493762263 | decreased | PMF | 133 | 21 | 6.65 | 102090 | 94763 | 5.77 | gi\|326514880 | |
| 72 | Clp protease ATP binding subunit | *Hordeum vulgare* | 2,07E-04 | 0,0494769 | 0,0208708 | 0,421829177 | decreased | PMF | 127 | 21 | 6.65 | 102090 | 94921 | 5.71 | gi\|326514880 | |
| 73 | Unidentified | | 3,65E-04 | 0,0153261 | | 0 | **decreased** | | | | | | 98302 | 5.93 | | |
| 74 | Phosphoribulokinase | *Triticum aestivum* | 0,00777089 | 1,11635 | 0,712787 | 0,638497783 | decreased | PMF | 201 | 55 | 5.72 | 45512 | 49203 | 5.15 | KPPR_WHEAT | |
| 75 | Unidentified | | 2,81E-04 | | 0,0586181 | | **INCREASED** | | | | | | 17868 | 6.53 | | |
| 76 | Translationally controlled tumor potein | *Hordeum vulgare* | 0,00224604 | 0,0208841 | 0,0517688 | 2,478861909 | INCREASED | MS/MS | 118 | 13 | 4.53 | 18929 | 26718 | 4.61 | TCTP_HORVU | |
| 77 | Unidentified | | 0,00129551 | 0,0102307 | 0,0787713 | 7,699502478 | INCREASED | | | | | | 24213 | 4.99 | | |
| 78 | Ricin-type beta-trefoil (carbohydrate-binding domain) | *Hordeum vulgare* | 1,51E-04 | 0,0312543 | 0,0855021 | 2,735690769 | INCREASED | PMF | 175 | 53 | 5.71 | 35765 | 43212 | 6.1 | gi\|326517467 | |
| 79 | Heat shock 70 kDa protein | *Hordeum vulgare* | 0,00501415 | 0,0200395 | 0,0552657 | 2,757838269 | INCREASED | PMF | 80 | 19 | 5.11 | 73301 | 86317 | 5.21 | gi\|326495158 | A15 |
| 80 | Unidentified | | 0,00473479 | | 0,0513186 | | **INCREASED** | | | | | | 15064 | 4.98 | | |
| 81 | Unidentified | | 0,0010757 | | 0,0188894 | | **INCREASED** | | | | | | 17080 | 4.92 | | |
| 82 | Unidentified | | 9,40E-04 | | 0,0245099 | | **INCREASED** | | | | | | 22070 | 5.52 | | |
| 83 | Unidentified | | 0,00284425 | | 0,0438718 | | **INCREASED** | | | | | | 31241 | 6.7 | | A13 |

| | | | | | | | | | | | | | | | | |
|---|---|---|---|---|---|---|---|---|---|---|---|---|---|---|---|---|
| 84 | Cysteine synthase | *Hordeum vulgare* | 2,39E-04 | | 0,0810459 | | **INCREASED** | PMF | 85 | 26 | 6.08 | 41129 | 41024 | 5.1 | gi\|326506704 | |
| 85 | Fructose-1,6-bisphosphate aldolase | *Hordeum vulgare* | 0,00136306 | 0,023501 | 0,0479504 | 2,04035573 | INCREASED | PMF | 114 | 31 | 6.08 | 39064 | 48742 | 6.78 | gi\|226316443 | A1 |
| 86 | Unidentified | | 0,005706 | 0,0274107 | | 0 | **decreased** | | | | | | 37590 | 5.36 | | |
| 87 | Thiamine pyrophosphate (TPP) family, transketolase subfamily | *Hordeum vulgare* | 2,64E-04 | 0,0100386 | | 0 | **decreased** | PMF | 118 | 34 | 5.45 | 74032 | 89697 | 5.46 | gi\|326533372 | |
| 88 | Unidentified | | 1,05E-05 | 0,0233682 | 0,0049353 | 0,211197268 | decreased | | | | | | 99788 | 5.37 | | |
| 89 | Heat shock 70 kDa protein, mitochondrial | *Phaseolus vulgaris* | 0,0143218 | 0,0543848 | 0,0332916 | 0,612148983 | decreased | MS/MS | 121 | 3 | 5.95 | 72721 | 75478 | 5.67 | HSP7M_PHAVU | A5 |
