# Supplementary material for: Analysis of Drought-Induced Proteomic and Metabolomic Changes in Barley (Hordeum vulgare L.) Leaves and Roots Unravels Some Aspects of Biochemical Mechanisms Involved in Drought Tolerance
Source: Front Plant Sci. 2016 Jul 26;7:1108. doi: 10.3389/fpls.2016.01108 (PMC4962459; doi:10.3389/fpls.2016.01108)
Supplement: Supplementary file 10 [file Table_5.PDF]

Table S5. List of drought-responsive proteins in Cam/B1/CI leaf extracts.

Spot no.	Protein name	Organism	ANOVA	Cam control	Cam stress	Stress/Control	Accumulation level	Method of identification	Score	% coverage	pI (theoretical)	Mw (theoretical)	Mw (experimental)	pI (experimental)	Accession number	Position on the gel
1	2-Cys peroxiredoxin BAS1, chloroplastic	*Hordeum vulgare*	0,00668025	0,0121968	0,0381006	3,123819362	INCREASED	PMF	82	40	5.4	23398	11494	6.44	BAS1_HORVU	A8
2	RNA recognition motif	*Hordeum vulgare*	1,63E-04	0,1423030	0,0387168	0,272072971	decreased	PMF	85	36	4.77	30719	32125	4.28	gi|326516784	
3	Glutathione S-transferase	*Hordeum vulgare*	0,00358229	0,0255268	0,0611039	2,393715624	INCREASED	PMF	112	34	8.91	33484	34009	5.61	gi|326507956	A7
4	Ubiquitin carboxyl-terminal hydrolase 19	*Arabidopsis thaliana*	0,00893555	0,0208164	0,0645235	3,099647393	INCREASED	MS/MS	28	1	4.8	77097	34662	5.16	UBP19_ARATH	A9
5	Oxygen-evolving enhancer protein 1, chloroplastic	*Solanum tuberosum*	0,0476194	0,519591	0,110131	0,211957097	decreased	MS/MS	312	13	5.84	35595	37353	5.28	PSBO_SOLTU	
6	Ferredoxin-NADP+ oxidoreductase	*Hordeum vulgare*	0,00998117	0,168052	0,0979795	0,583030848	decreased	PMF	101	39	8.29	39971	40252	6.37	gi|326492141	A10
7	Ferredoxin-NADP+ oxidoreductase	*Triticum aestivum*	0,0021909	0,20178	0,248442	1,231251858	INCREASED	PMF	123	48	6.92	40491	40252	5.65	gi|20302473	
8	Unidentified		0,0307113	0,013688	0,0364749	2,664735535	INCREASED						41337	6.17		
9	Unidentified		0,00162949	0,0711978	0,183571	2,578324049	INCREASED						40869	5.16		A14
10	Unidentified		0,216608	0,12827	0,165565	1,290753879	INCREASED						43222	6.72		
11	Unidentified		2,35E-04	0,055791	0,10314	1,848685272	INCREASED						44377	6.04		
12	Ribulose bisphosphate carboxylase/oxygenase activase B, chloroplastic	*Triticum aestivum*	5,64E-04	0,011835	0,185658	15,68719899	INCREASED	PMF	145	41	6.92	48012	47387	5.58	gi|7960277	A3
15	Phenylalanine ammonia-lyase	*Bromheadia finlaysoniana*	7,68E-05	0,0508611	0,0225233	0,44283942	decreased	MS/MS	39	2	7.1	76605	65229	5.05	PALY_BROFI	
16	Glutamate dehydrogenase	*Hordeum vulgare*	0,00599432	0,0273513	0,106122	3,87996183	INCREASED	PMF	86	27	5.33	54461	66998	5.6	gi|326519016	
17	Enolase	*Hordeum vulgare*	6,68E-04	0,220053	0,279485	1,27008039	INCREASED	PMF	132	39	5.39	48601	69269	5.6	gi|326490934	A4
18	Glutathione reductase, chloroplastic	*Hordeum vulgare*	0,0054361	0,0236746	0,0323491	1,366405346	INCREASED	PMF	87	28	7.64	59615	72942	6.04	gi|157362217	
20	ATP synthase subunit alpha, chloroplastic	*Hordeum vulgare*	0,0179638	0,0308022	0,048189	1,564466174	INCREASED	PMF	76	19	6.32	55317	76683	5.93	ATPA_HORVU	
21	Ferredoxin-nitrite reductase, chloroplastic	*Hordeum vulgare*	3,33E-04	0,0832444	0,0336241	0,403920264	decreased	PMF	122	20	6.33	66660	78493	6.3	gi|326505210	A12
23	Heat shock protein 70 kDa, mitochondrial	*Phaseolus vulgaris*	0,00928046	0,0435198	0,0574036	1,319022606	INCREASED	MS/MS	121	3	5.95	72721	82794	5.47	HSP7M_PHAVU	A5
24	Heat shock protein 70 kDa, chloroplastic	*Hordeum vulgare*	9,86E-04	0,157451	0,0856052	0,543694229	decreased	PMF	120	21	5.04	73955	85601	4.77	gi|326492960	A6
25	Ricin-type beta-trefoil lectin domain-like	*Hordeum vulgare*	0,0101948	0,0429553	0,0786682	1,831396824	INCREASED	PMF	122	39	5.71	35765	43542	6.15	gi|326517467	
26	Fructose-1,6-bisphosphate aldolase	*Hordeum vulgare*	5,39E-04		0,0556374		**INCREASED**	PMF	114	31	6.08	39064	48286	6.81	gi|226316443	A1
27	Unidentified		0,00176135	0,00614121	0,0212415	3,458846058	INCREASED						74416	6.17		
28	Heat shock protein 70 kDa, chloroplastic	*Brachypodium distachyon*	0,0037359	0,16762	0,12759	0,761186016	decreased	PMF	107	25	5.04	73203	84890	4.7	gi|357134135	
29	Heat shock protein 70 kDa, chloroplastic	*Spinacia oleracea*	0,0259503	0,134993	0,0661696	0,490170601	decreased	MS/MS	108	4	4.87	64918	85458	4.73	HSP7S_SPIOL	
30	Unidentified		9,70E-04	0,04727	0,117498	2,48567802	INCREASED						30460	5.15		
33	Unidentified		0,00731553		0,0421895		**INCREASED**						31162	6.76		A13
34	Ascorbate peroxidase	*Hordeum vulgare*	1,68E-04		0,147918		**INCREASED**	PMF	117	49	5.10	27964	32907	5.16	gi|15808779	
36	Glyceraldehyde-3-phosphate dehydrogenase A, chloroplastic	*Zea mays*	1,53E-04		0,0194159		**INCREASED**	MS/MS	40	2	7.00	43182	44377	6.35	G3PA_MAIZE	
37	Unidentified		6,55E-04		0,0419277		**INCREASED**						44716	5.91		
38	L-idonate 5-dehydrogenase	*Hordeum vulgare*	5,55E-09		0,040462		**INCREASED**	PMF	199	58	6.27	39564	54434	6.79	gi|326515958	
39	Glutamine synthetase leaf isozyme, chloroplastic	*Hordeum vulgare*	1,57E-04	0,936783	2,30833	2,464103213	INCREASED	PMF	119	40	5.75	46902	53295	5.1	gi|755762	A11
40	Ribulose bisphosphate carboxylase/oxygenase activase A, chloroplastic	*Hordeum vulgare*	4,91E-04		0,134756		**INCREASED**	PMF	194	39	8.04	51383	57456	5.14	RCAA_HORVU	
42	Glutamine synthetase leaf isozyme, chloroplastic	*Hordeum vulgare*	0,00759593	0,0872495		0	**decreased**	MS/MS	243	7	5.11	47406	31360	5.01	GLNA2_HORVU	
44	Clp protease ATP binding subunit	*Hordeum vulgare*	2,82E-02	0,0557778	0,0767487	1,375972161	INCREASED	PMF	127	21	6.65	102090	93196	5.77	gi|326514880	
46	Ascorbate peroxidase	*Hordeum vulgare*	3,57E-02	0,0133261	0,0381987	2,866457553	INCREASED	PMF	80	34	5.85	27532	35670	5.21	gi|3688398	
47	Betaine aldehyde dehydrogenase	*Hordeum vulgare*	1,21E-03	0,0234434	0,059385	2,533122329	INCREASED	PMF	85	19	5.47	55276	68540	5.13	gi|15147871	
48	Ribulose bisphosphate carboxylase/oxygenase large subunit-binding protein subuni	*Secale cereale*	5,62E-03	0,440628	0,319764	0,725700591	decreased	MS/MS	376	11	4.7	53721	68900	5.65	RUBB_SECCE	A2
49	Clp protease ATP binding subunit	*Hordeum vulgare*	1,54E-02	0,0266365	0,0370663	1,391560453	INCREASED	PMF	127	21	6.65	102090	102889	6.44	gi|326514880	
50	Heat shock protein 70 kDa	*Hordeum vulgare*	3,78E-03	0,0070332	0,0405656	5,767730194	INCREASED	PMF	80	19	5.11	73301	79546	5.14	gi|326495158	A15
